# The combination of Ephedrae herba and coixol from Coicis semen attenuate adiposity via glucocorticoid receptor regulation

**DOI:** 10.1038/s41598-023-47553-3

**Published:** 2023-11-21

**Authors:** Ga-Ram Yu, Jai-Eun Kim, Dong-Woo Lim, Won-Hwan Park

**Affiliations:** 1 Department of Diagnostic, College of Korean Medicine, Dongguk University, Goyang, 10326 Republic of Korea; 2 Department of Pathology, College of Korean Medicine, Dongguk University, Goyang, 10326 Republic of Korea; 3 Institute of Korean Medicine, Dongguk University, Goyang, 10326 Republic of Korea

**Keywords:** Drug development, Molecular medicine, Endocrine system and metabolic diseases

## Abstract

The enhanced therapeutic effects and mechanisms of certain herbal combination in various herbal prescriptions are mostly unclear. A combination of two herbs, namely Ephedrae herba (EH) and Coicis semen (CS), has been commonly prescribed for obesity. In our previous work, the combination of EH and CS was studied using network pharmacological approach to predict its pharmacological targets and in vitro experiments to evaluate its efficacy on obesity. Although we demonstrated enhanced anti-adiposity effects of the combination on matured adipocytes, the molecular mechanisms and contributing compounds underlying the effects of EH-CS combination on adiposity or adipogenesis were not fully elucidated. The current study adopted integrated bioinformatics analysis to precisely validate potential targets of EH-CS by screening differentially expressed genes (DEGs) of morbid obesity patients from NCBI gene expression omnibus (GEO). Based on the functional cluster analysis of down-regulated DEGs, the anti-adipogenesis mechanism of EH-CS combination was speculated with KEGG enrichment analysis. Furthermore, we investigated the combinational effects of EH and coixol, or stigmasterol, the two compounds in CS which were expected to have main beneficial effects in metabolic diseases. Moreover, distinct effect of the combination on transcriptional activity of glucocorticoid receptor (GR) was investigated using electrophoretic mobility shift assay (EMSA). The EH-CS combination was predicted to modulate down-regulated genes which are involved in KEGG pathways crucial to metabolic disease in morbidly obese individuals. The combination of EH with CS compounds significantly increased the phosphorylation of acetyl-coA carboxylase (ACC), AMP-activated protein kinase (AMPK), and protein kinase B (AKT) in 3T3-L1 cells and decreased intracellular lipid accumulation. The two CS compounds significantly increased the anti-adipogenesis/lipogenesis effects of EH by inhibiting the gene expression levels. Finally, the combination of EH and coixol inhibited dexamethasone-induced GR translocation to the nucleus and transcriptional binding activity in adipocytes. The combination of EH and CS could be considered a therapeutic strategy for treating metabolic diseases, including obesity.

## Introduction

Obesity is a complex multifactorial disease defined by excessive fat accumulation. It is associated with co-morbid conditions such as hypertension and dyslipidemia and increases the risk of diseases, such as type 2 diabetes (T2D), cardiovascular disease (CVD), and cancer^1^. Several factors which increase energy intake and decrease energy expenditure work in combination, resulting in energy excess leading to the development of obesity^2^. The process by which adipocytes develop and accumulate as obese adipose tissue is referred to as adipogenesis and the major mechanisms of adipogenesis are adipocyte hypertrophy and hyperplasia^3^. Adipogenesis is mainly regulated by various adipogenic transcription factors such as peroxisome proliferator-activated receptor gamma (PPARγ) and CCAAT/enhancer binding protein α (C/EBP alpha)^4^.

Glucocorticoids (GCs) are important regulators of lipid metabolism, which bind to the glucocorticoid receptor (GR). The GR functions as a transcription factor to exert pleiotropic effects^5,6^. The GR resides in the cytosol complex with a variety of proteins including heat shock protein 90 (hsp90), heat shock protein 70 (hsp70) and the protein FKBP4 (FK506-binding protein), in the absence of GCs. Upon binding with the GCs the GR complex becomes dissociated from heat shock protein 90 and is transferred to the nucleus^7^. Consequently, homodimerization of GR occurs on the glucocorticoid response elements (GREs) in the promoter region for transcriptional activity^8^. This has a fundamental impact on adipose tissue physiology and obesity by modulating adipogenesis^5^. For instance, GR promotes adipogenesis by directly promoting the expression of adipogenic transcription factors such as C/EBPα and PPARγ during the early stages of differentiation^9^. GR can modulate metabolic disorders such as obesity, lipid abnormality, and insulin resistance by altering lipid metabolism and insulin sensitivity in adipocytes^10^.

Although the mechanism by which the GCs induce obesity (especially on the directionality of effect by their duration of treatment) is still a subject of controversy, it is generally accepted that the presence of GCs, such as dexamethasone, is necessary for the initiation or acceleration of adipogenesis in preadipocytes by binding to GR^11^. Therefore, the antagonization of GRs can be a potential strategy for treating obesity as was demonstrated in the case of mifepristone^12,13^.

Herbal medicine is an extensive natural resource of medicinal compounds, and herbal resources are generally regarded as a good repository for identifying effective and safe drugs^14^. In traditional Korean medicine, the exceptional concept of herb-herb interaction is utilized when prescriptions are formulated. Herbal combinations provide enhanced pharmacological properties and safety profiles compared to a single herb and are considered a critical therapeutic strategy for treating diseases or syndromes. However, their detailed pharmacological mechanisms are largely unknown^15^. The ‘one drug, one target’ approach has evident shortcoming in interpreting the pharmacological mechanisms of multi-herb prescriptions^16^.

The network pharmacology (NP) approach provides a valuable novel methodology for understanding drug combinations for the treatment of complex diseases^17^. The NP approach, based on molecular information from herbal databases, allows us to understand the complex molecular mechanisms of herbal pairs and to predict their efficacy against diseases of interest. Transcriptomics profiling can provide researchers with a deeper insight into the pathophysiology of certain diseases or phenotypes by screening the gene expression in its entirety^18^. Interpretation of the high-throughput gene expression data, represented as differentially expressed genes (DEGs), may unravel significant therapeutic targets (including genes, pathways, etc.) for treating diseases of interest^19^. Together, the NP approach and transcriptomics profiling can be integrated and used to decipher the mode of action of herbal medicine with a fortified target background relevant to specific diseases. For instance, Ma et al. extracted meaningful gene sets of human hepatocellular carcinoma (HCC) from a public microarray database to compare the outputs with the therapeutic targets of baicalein, which is a major compound of *Scutellaria baicalensis*^20^. In a recent study, the bioinformatics analysis results of DEGs obtained from the microarray profiling data and the NP analysis technique were used to inversely suggest herbal medicine candidates to treat obesity^21^.

A previous review paper questioned the possibilities of a drug interaction between two herbs, namely Ephedrae herba (EH) and Coicis semen (CS), in their frequent use against obesity in clinics practicing Korean medicine^22^. However, the study was a literature review and did not provide experiment based evidences for the herbal pair. In another study, it had been speculated that the pair is responsible for the main pharmaceutical action in two distinct herbal prescriptions with similar weight-reducing efficacy being part of herbal medicines with distinctly different compositions^23^. As is well known, the hot water extract of EH contains a significant amount of ephedrine which has been known to exert a weight-loss effect with sympathomimetic properties^24^. On the other hand, several studies have reported the anti-obesity efficacy of CS. However, the reported mechanism was different from that of EH^25^.

In our earlier study, the herbal pair of EH and CS was evaluated for their effect on obesity^25^. Five ratios of the EH and CS mixture (0:100, 25:70, 50:50, 75:25, and 100:0 [w/w]) were evaluated for their anti-adipogenesis effects on 3T3-L1 preadipocytes. The 50:50 EH-CS combination attenuated lipid accumulation and adipogenic gene expression significantly while increasing the phosphorylation of AMP-activated protein kinase (AMPK), with relatively low inflammatory profiles in palmitic acid-induced inflamed preadipocytes^25^. However, the authors reported the limited efficacy of the CS extract alone on adipogenesis as compared to the significant effects of the mixed extract or the EH extract alone. This could suggest the indirect pharmaceutical action of CS in attenuating adipogenesis. The key synergistic mechanism or principal molecule of EH-CS acting against adipogenesis remained unidentified. In particular, the key compound responsible for the improved efficacy of the herbal pair was not discussed and even the possibility of a pair of bioactive compounds was just speculated from the network pharmacological analysis and other literature^25^.

In the present study, we investigated the pharmacological mechanism of the chosen ratio of the EH-CS combination on adipogenesis in mature adipocytes. Furthermore, the CS-derived compounds, coixol and stigmasterol were investigated in combination with the EH extract to assess their combined anti-adipogenesis effects. Specifically, the impact of the combination on the transcriptional activity of GR was also investigated using various methods.

## Results

### Validation of gene expression omnibus (GEO) human microarray data and isolation of DEGs overlapping with potential targets of EH-CS

Microarray gene expression data of GSE59034 were obtained from the GEO database and further processed at GEO2R using its built-in web tool. The boxplot of the 32 samples revealed well-normalized gene expression values of all the samples (Fig. S1A). Of the total of 33,297 genes analyzed, 9598 genes were sorted as DEGs (Fig. S1B), while the histogram showed a skewed distribution of adjusted p-values between the two groups (Fig. S1C). A uniform manifold approximation and projection for dimension reduction (UMAP) plot showed a relatively intimate distance in the reduced Euclidean dimension between samples from identical groups (Lean vs Obese) (Fig. S1D). The volcano plot showed the distribution of DEGs arranged by their adjusted p-values and log2 fold changes (Fig. S1E).

Of these DEGs, duplicate genes were removed and 6621 genes were included as the final list of DEGs (Fig. S2). Among the DEGs associated with human obesity, the potential target DEGs for the action of EH and CS were analyzed. Consequently, a total of 197 DEGs were isolated as potential targets of the EH-CS combination, of which 123 genes were involved in up-regulated genes and 74 in down-regulated genes (Fig. S3A,B).

### Functional clustering of potential targets from EH-CS

A total of 123 up-regulated DEGs associated with morbid obesity, which were also potential targets of EH-CS were clustered into three functional groups using the MCODE algorithm (Fig. S4). However, the target lists were unequally distributed with a focus on EH targets, and not on EH-CS targets. In addition, the top enriched biological process (BP) terms and KEGG pathways for the up-regulated functional clusters were not highly correlated with metabolic diseases or pathways.

**Figure 1:**
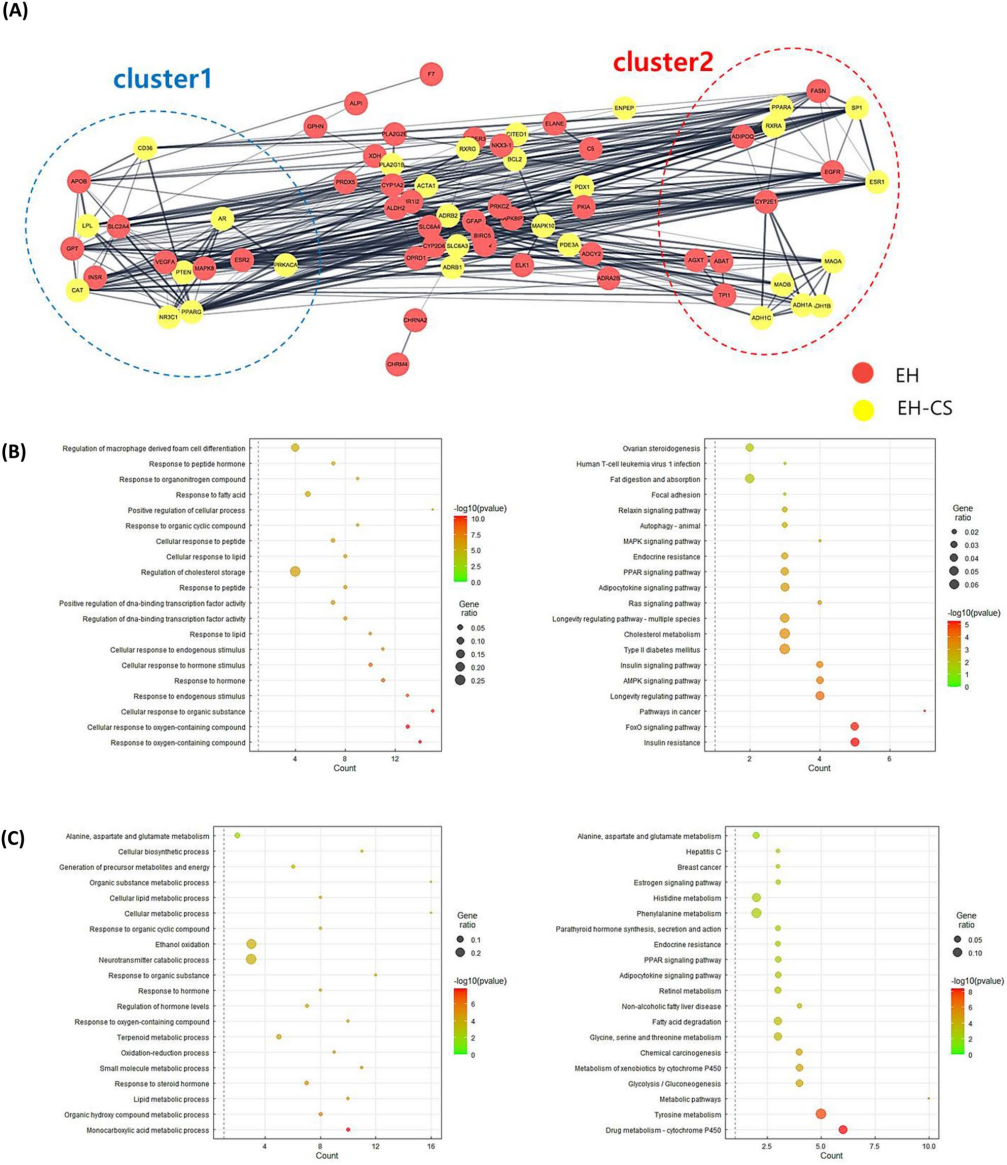
Clustering of the PPI network and enrichment analysis of obesity-related down-regulated DEGs overlapping with the EH-CS combination. (**A**) Total PPI network and functional clusters of down-regulated DEGs. (**B**,**C**) Bubble plot visualization of enrichment analysis (BP terms and KEGG pathways) from two functional clusters of the herbal combination (B for cluster1, C for cluster2). BP term (left) and KEGG pathways (right). *PPI* protein–protein interaction, *DEGs* differentially expressed genes, *BP* biological process, *KEGG* Kyoto encyclopedia of genes and genomes.

Also, 74 down-regulated DEGs, except for 4 isolated genes from PPI (protein–protein interaction) -network, were clustered into two functional clusters (Fig. 1A). The down-regulated DEGs were notably affected by both EH and CS, which reveals the co-regulation of these common genes by the two herbs (Table 1). The first cluster of down-regulated DEGs was highly enriched with the BP terms of ‘response to oxygen-containing compound’, ‘cellular response to organic substances’, ‘response to endogenous stimulus’, and ‘response to hormone’ etc. (Fig. 1B). In addition, the cluster was also shown to be heavily related to metabolism-related pathways such as ‘insulin resistance’, ‘FoxO signaling pathway’, and ‘AMPK signaling pathway’.

**Table 1:** Down-regulated DEGs overlapping with potential targets of the EH-CS combination, identified from subcutaneous adipose tissue originating from morbidly obese patients.

Down-regulated DEGs overlapping with EH (45)			Down-regulated DEGs overlapping with EH-CS (29)		
Genes	Log2 (FC)	(− Log10 (p value))	Genes	Log2 (FC)	(− Log10 (p value))
ABAT	− 0.219	1.937	ACTA1	− 0.331	3.392
ADCY2	− 0.381	2.991	ADH1A	− 0.784	8.158
ADIPOQ	− 0.239	6.453	ADH1B	− 0.748	6.819
ADRA2B	− 0.176	2.029	ADH1C	− 0.713	7.829
ADSSL1	− 0.864	5.429	ADRB1	− 0.352	1.966
AGXT	− 0.176	2.006	ADRB2	− 0.193	2.909
ALDH2	− 0.297	6.312	AR	− 0.199	2.396
ALPI	− 0.161	1.905	BCL2	− 0.237	2.857
APOB	− 1.441	5.99	CAT	− 0.118	1.972
ATF2	− 0.219	5.601	CD36	− 0.088	2.382
BIRC5	− 0.128	2.187	CITED1	− 0.161	1.949
C5	− 0.406	2.654	ENPEP	− 0.331	2.94
CHRM4	− 0.216	1.936	ESR1	− 0.738	6.589
CHRNA2	− 0.236	2.35	LPL	− 0.236	4.281
CYP1A2	− 0.267	2.417	MAOA	− 0.404	5.464
CYP2D6	− 0.355	4.929	MAOB	− 0.566	6.707
CYP2E1	− 0.179	2.697	MAPK10	− 0.212	1.854
DCAF5	− 0.147	2.867	NR3C1	− 0.37	5.82
EGFR	− 0.357	5.689	PDE3A	− 0.859	5.712
ELANE	− 0.325	3.446	PDX1	− 0.171	2.042
ELK1	− 0.266	3.906	PLA2G1B	− 0.212	3.73
ESR2	− 0.534	4.108	PPARA	− 0.298	2.958
F7	− 0.177	2.22	PPARG	− 0.308	4.825
FASN	− 0.646	3.402	PRKACA	− 0.168	1.845
GFAP	− 0.208	2.505	PTEN	− 0.34	6.33
GPHN	− 0.447	4.059	RXRA	− 0.21	2.78
GPT	− 0.493	3.853	RXRG	− 0.331	3.237
INSR	− 0.302	4.189	SLC6A3	− 0.32	3.733
IVL	− 0.304	3.807	SP1	− 0.172	3.599
MAPK8	− 0.171	1.939			
MAPK8IP2	− 0.234	1.843			
NKX3-1	− 0.226	2.428			
NPEPPS	− 0.225	3.477			
NR1I2	− 0.105	1.947			
OPRD1	− 0.285	3.409			
PKIA	− 0.217	2.095			
PLA2G2E	− 0.223	3.035			
PRDX5	− 0.171	2.364			
PRKCZ	− 0.205	2.453			
PTGER3	− 0.239	3.364			
SLC2A4	− 0.987	6.674			
SLC6A4	− 0.333	4.315			
TPI1	− 0.231	2.336			
VEGFA	− 0.467	3.066			
XDH	− 0.287	4.811			

*DEGs* differentially expressed genes, *EH-CS* Ephedrae herba-Coicis semen.

The second functional cluster was enriched with the BP terms of ‘monocarboxylic acid metabolic process’, ‘lipid metabolic process’, and ‘response to steroid hormones’ (Fig. 1C). Also, the cluster had a high interaction with metabolism-related KEGG pathways such as ‘drug metabolism-CYP450’, ‘tyrosine metabolism’, and ‘metabolic pathways’.

### EH-CS attenuated lipid accumulation in mature 3T3-L1 cells and increased AMPK and AKT signaling

**Figure 2:**
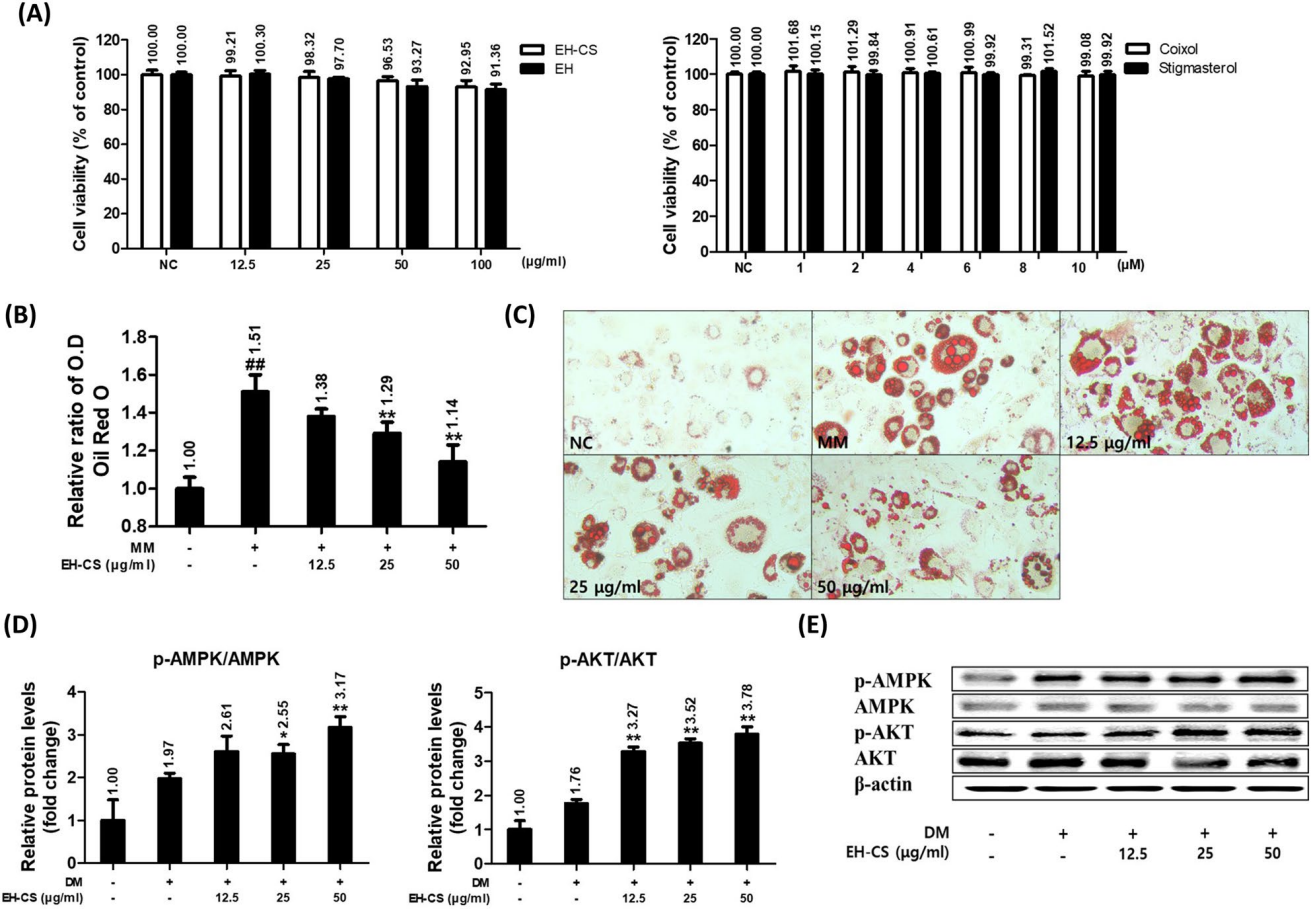
EH-CS inhibited lipid accumulation and up-regulated phosphorylation of AMPK and AKT in 3T3-L1 cells. (**A**) 3T3-L1 cells were incubated for 24 h with samples, including EH-CS (0–100 μg/ml), Coixol (0–10 μM), and Stigmasterol (0–10 μM). Cell viability was determined using an EZ-cytox assay kit. (**B**,**C**) Mature 3T3-L1 cells were produced by treating 3T3-L1 preadipocytes with a differentiation medium for 3 days and then treating them with a maturation medium for 8 days. Matured 3T3-L1 cells were co-treated with EH-CS and the maturation medium for 8 days. Lipid accumulation was determined using an Oil Red O (ORO) staining assay. (**D**,**E**) 3T3-L1 cells were incubated in a differentiation medium with EH-CS at 12.5, 25, and 50 μg/ml for 24 h. Relative phosphorylation of AMPK and AKT expressions as determined by Western blot. Band intensities were measured densitometrically and divided by that of non-phosphorylated AMPK and AKT. Results are presented as the means ± SDs of three independent experiments. ^##^*P* < 0.1 versus preadipocytes, and *P < 0.05, **P < 0.01 versus maturated adipocytes. *EH-CS* Ephedrae herba-Coicis semen, *AMPK* AMP-activated protein kinase (AMPK), *AKT* protein kinase B.

The 3T3-L1 cell line is widely adopted as in vitro research model of adipogenesis to evaluate the anti-obesity effect of drugs. The cell line differentiates into an adipocyte-like phenotype under appropriate stimulation^26–28^. The cytotoxicity of EH-CS, EH, stigmasterol, and coixol was evaluated. The mature 3T3-L1 cells were incubated with various concentrations of the samples for 24 h. The cells did not show any significant decrease in viability at EH-CS or EH concentrations of < 100 μg/ml (92.95% and 91.36% at 100 μg/ml respectively). Coixol or stigmasterol at 10 μM did not have any significant cytotoxic effects (99.08% and 99.92% at 10 μM respectively) (Fig. 2A). Subsequent experiments were performed using EH-CS (12.5, 25, 50 μg/ml). Oil Red O staining (ORO) showed that the EH-CS treatment at 25, 50 μg/ml for 8 days significantly inhibited the development of lipid droplets and lipid accumulation in mature adipocytes (Fig. 2B and C). Furthermore, we investigated the activation of AMP-activated protein kinase (AMPK) and protein kinase B (AKT) associated with energy metabolism in 3T3-L1 cells. The phosphorylation of AMPK and AKT was dose-dependently increased by the EH-CS treatment (Fig. 2D and E).

### EH-CS alleviated markers related to adipogenesis/lipogenesis in 3T3-L1 cells

**Figure 3:**
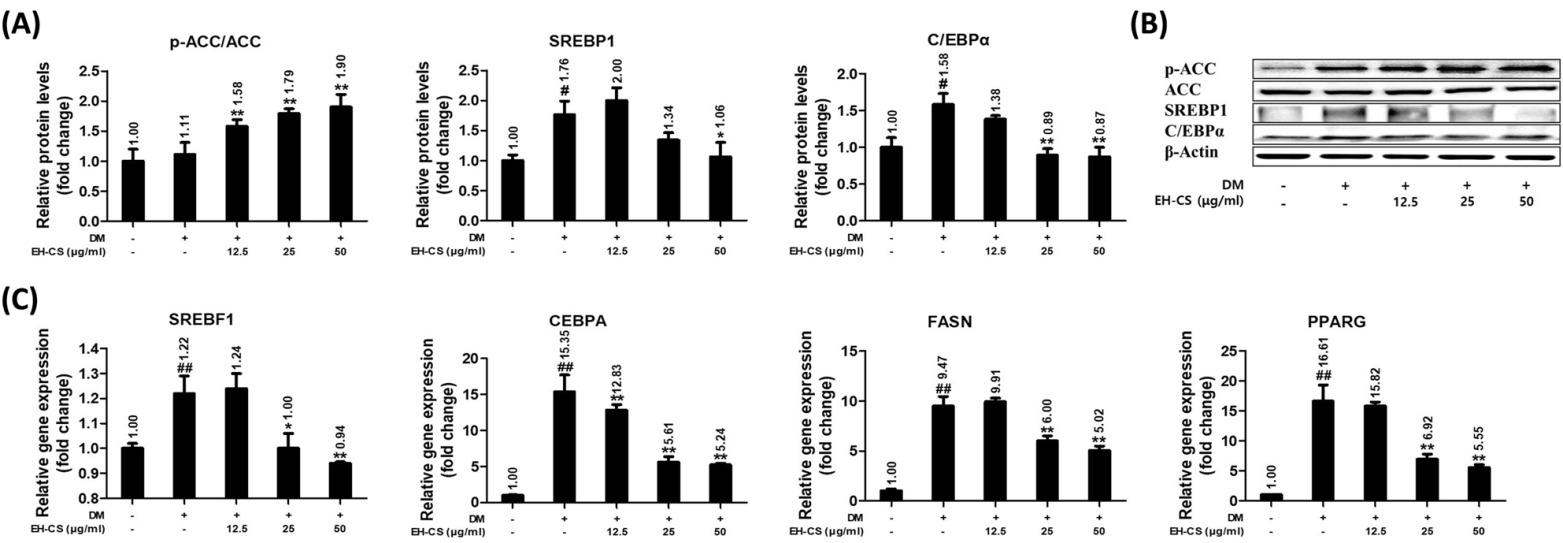
EH-CS regulated the relative protein and gene expression levels of adipogenesis/lipogenesis-associated markers in 3T3-L1 cells. 3T3-L1 cells were incubated in a differentiation medium with EH-CS at 12.5, 25, and 50 μg/ml for 12 or 24 h. (**A**,**B**) Relative protein expression levels of ACC, SREBP1, and C/EBPα as determined by the Western blot. (**C**) Relative gene expression levels of SREBP1, CEBPA, FASN, and PPARγ as determined by real-time quantitative PCR. Results are presented as the means ± SDs of three independent experiments. ^#^*P* < 0.05, ^##^*P* < 0.01 versus preadipocytes, and *P < 0.05, **P < 0.01 versus maturated adipocytes. *EH-CS* Ephedrae herba-Coicis semen, *ACC* acetyl-coA carboxylase, *SREBP1* sterol regulatory binding protein, *C*/*EBPα* CCAAT/enhancer binding protein α, *FASN* fatty acid synthase, *PPARγ* peroxisome proliferator-activated receptor gamma.

The inhibitory effects of EH-CS on adipogenesis/lipogenesis in 3T3-L1 cells were investigated using the Western blot or qPCR. At a concentration of 50 μg/ml, EH-CS significantly increased the phosphorylation of acetyl-CoA carboxylase (ACC) (Fig. 3A and B). The maturation of the 3T3-L1 cells markedly elevated the sterol regulatory binding protein 1 (SREBP1) and C/EBPα expressions. However, treatment with EH-CS for 24 h significantly inhibited the protein and gene expression of SREBP1 and C/EBPα dose-dependently. Under the same conditions, EH-CS significantly suppressed the gene expression of fatty acid synthase (FASN) and peroxisome proliferator-activated receptor gamma (PPAR γ) (Fig. 3C).

### Combination of EH and coixol significantly attenuated lipid accumulation and activated phosphorylation of AMPK and AKT in 3T3-L1 cells

**Figure 4:**
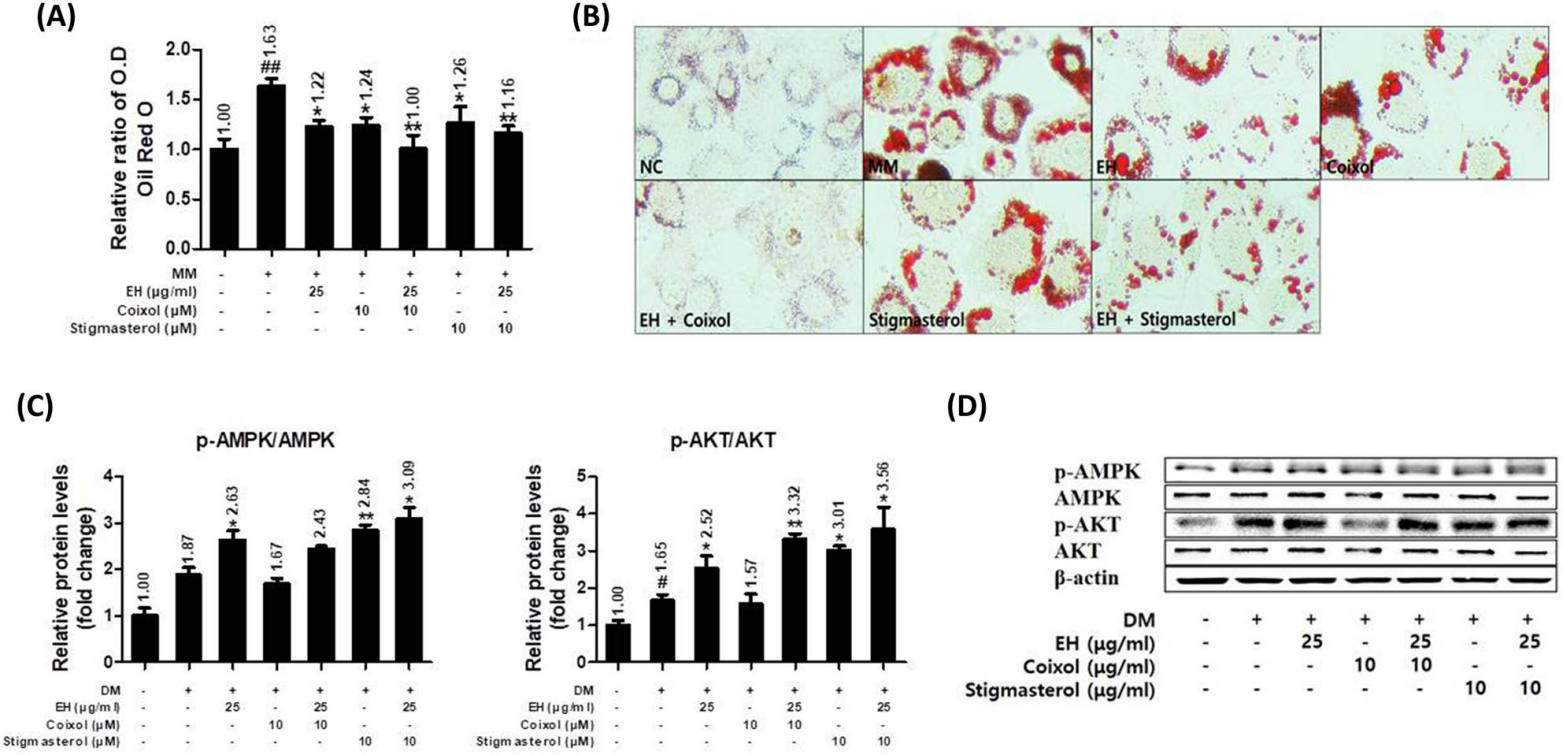
Coixol and Stigmasterol up-regulated the anti-adipogenesis/lipogenesis effects of EH and the activation of AMPK and AKT in 3T3-L1 cells. (**A**,**B**) Matured 3T3-L1 cells were co-treated with the samples and maturation medium for 8 days. Lipid accumulation was determined using an ORO assay. (**C**,**D**) Relative phosphorylation of AMPK and AKT expressions as determined by Western blot. Results are presented as the means ± SDs of three independent experiments. ^#^*P* < 0.05, ^##^*P* < 0.01 versus preadipocytes, and *P < 0.05, **P < 0.01 versus maturated adipocytes. *EH* Ephedrae herba, *AMPK* AMP-activated protein kinase (AMPK), *AKT* protein kinase B.

The combination treatment of EH (25 μg/ml) with either coixol (10 μM) or stigmasterol (10 μM) for 8 days significantly inhibited adipocyte maturation, as demonstrated by the loss of lipid accumulation in Oil Red O staining. EH with coixol treatment most significantly reduced lipid accumulation to levels similar to that of the untreated preadipocytes (Fig. 4A and B). EH with stigmasterol increased the phosphorylation of AMPK and AKT the most, compared to treatment with each of them alone. Meanwhile, coixol (10 μM) treatment alone did not affect the phosphorylation of AMPK and AKT, but coixol with EH treatment promoted the phosphorylation of AKT more than the EH treatment alone (Fig. 4C and D).

### Combination of EH and coixol reduced markers related to adipogenesis/lipogenesis in 3T3-L1 cells

**Figure 5:**
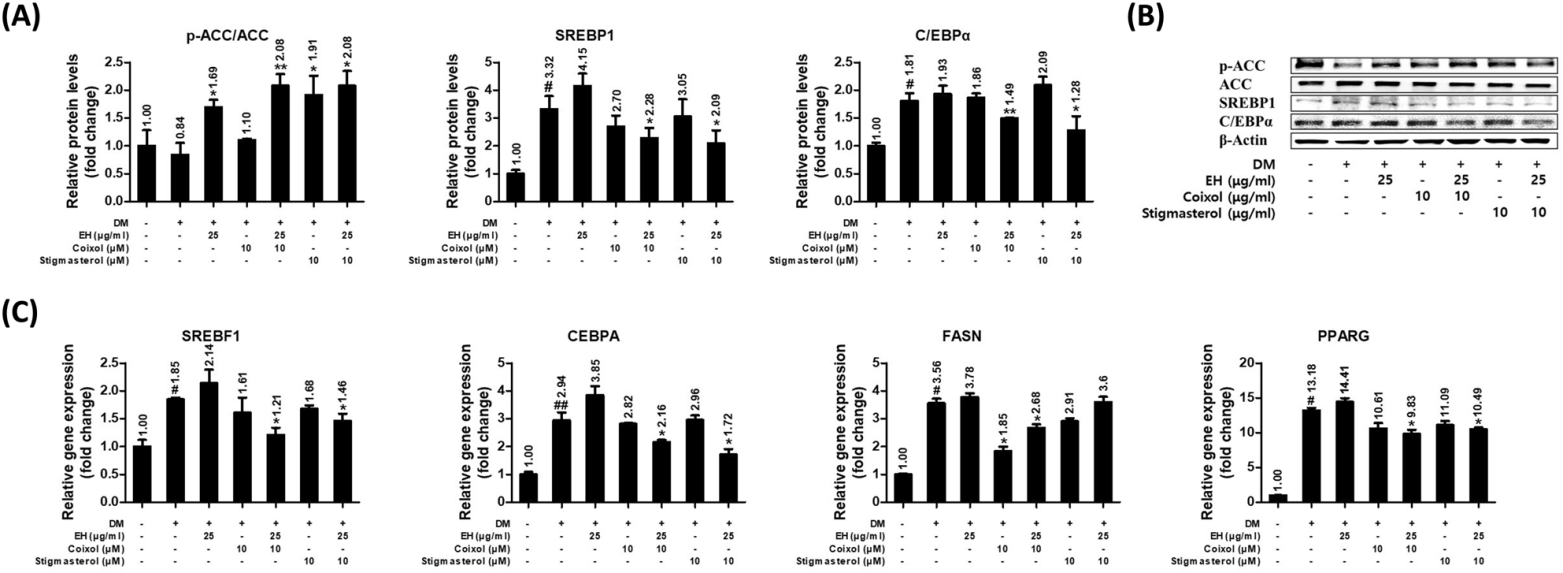
Coixol and Stigmasterol up-regulated the anti-adipogenesis/lipogenesis effects of EH. (**A**,**B**) Relative protein expression levels of ACC, SREBP1, and C/EBPα as determined by Western blot. (**C**) Relative gene expression levels of SREBP1, CEBPA, FASN, and PPARγ as determined by real-time quantitative PCR. Results are presented as the means ± SDs of three independent experiments. ^#^*P* < 0.05, ^##^*P* < 0.01 versus preadipocytes, and *P < 0.05, **P < 0.01 versus maturated adipocytes. *ACC* acetyl-coA carboxylase, *SREBP1* sterol regulatory binding protein, *C/EBPα* CCAAT/enhancer binding protein α, *FASN* fatty acid synthase, *PPARγ* peroxisome proliferator-activated receptor gamma, *EH* Ephedrae herba.

The inhibitory effects of EH with coixol or stigmasterol in combination on adipogenesis/lipogenesis in 3T3-L1 cells were investigated using the Western blot or qPCR. EH with coixol treatment increased the phosphorylation of ACC more significantly than the EH treatment alone. However, the effect of stigmasterol on the phosphorylation of ACC was not enhanced by EH which did not affect SREBP1 and C/EBPα expressions. But the combined treatment of EH with either coixol or stigmasterol significantly reduced protein or gene expressions of SREBP1 and C/EBPα (Fig. 5A and B). In addition, the combination of EH and coixol reduced the gene expression of FASN and PPARγ more than the combination of EH and stigmasterol (Fig. 5C).

### EH with coixol and stigmasterol in combination blocked the nuclear translocation of GR and SREBP1 in matured 3T3-L1 cells

**Figure 6:**
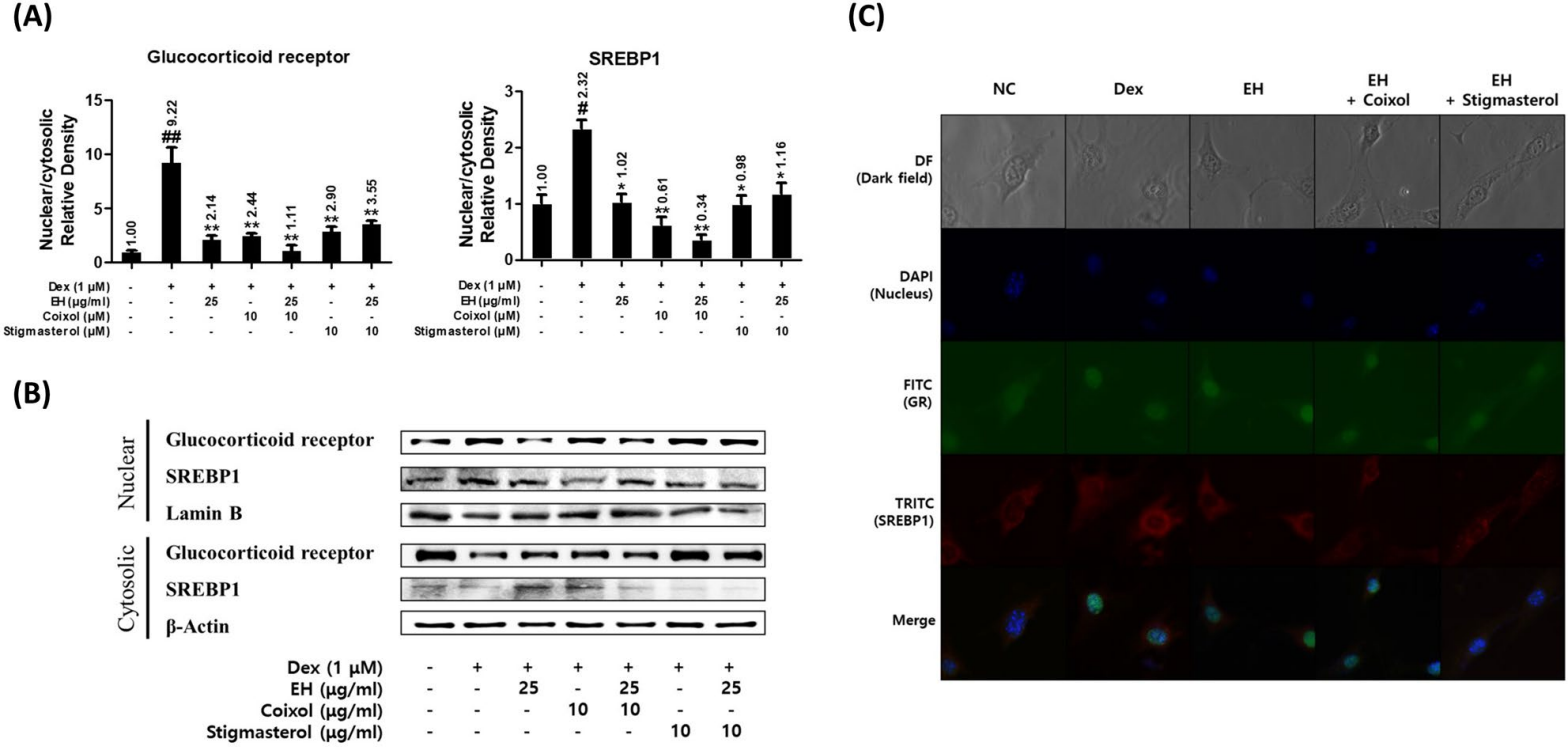
EH, coixol, and stigmasterol block the nuclear translocation of GR and SREBP1 in dexamethasone-induced 3T3-L1 cells. (**A**,**B**) Nuclear translocation of GR and SREBP1 as determined by Western blot. Each nuclear- or cytosolic-glucocorticoid receptor level was divided by Lamin B level. Further, the adjusted nuclear GR level was divided by the cytosolic level. (**C**) The translocation of transcription factors was visualized by co-immunofluorescence microscopy. Results are presented as the means ± SDs of three independent experiments. ^#^*P* < 0.05, ^##^*P* < 0.01 versus adipocytes, and *P < 0.05, **P < 0.01 versus dexamethasone-induced adipocytes. *GR* glucocorticoid receptor, *SREBP1* sterol regulatory binding protein, *EH* Ephedrae herba.

The inhibitory effects of EH with coixol or stigmasterol in combination on the nuclear translocation of GR and SREBP1 in matured 3T3-L1 cells were investigated using the Western blot or immunofluorescence microscopy. The Western blot showed that dexamethasone increased the translocation of GR and SREBP1 in matured 3T3-L1 cells. EH significantly blocked GR and SREBP1 translocations to the nucleus, and the additional coixol treatment enhanced the inhibitory effect (Fig. 6A and B). However, EH combined with stigmasterol had no additional effect on the inhibition of GR and SREBP1 activation. The immunofluorescence images also supported the localization of GR and SREBP1 in the nucleus (Fig. 6C).

### Coixol blocks nuclear GR binding activities to glucocorticoid response elements (GREs) in dexamethasone-stimulated 3T3-L1 cells

**Figure 7:**
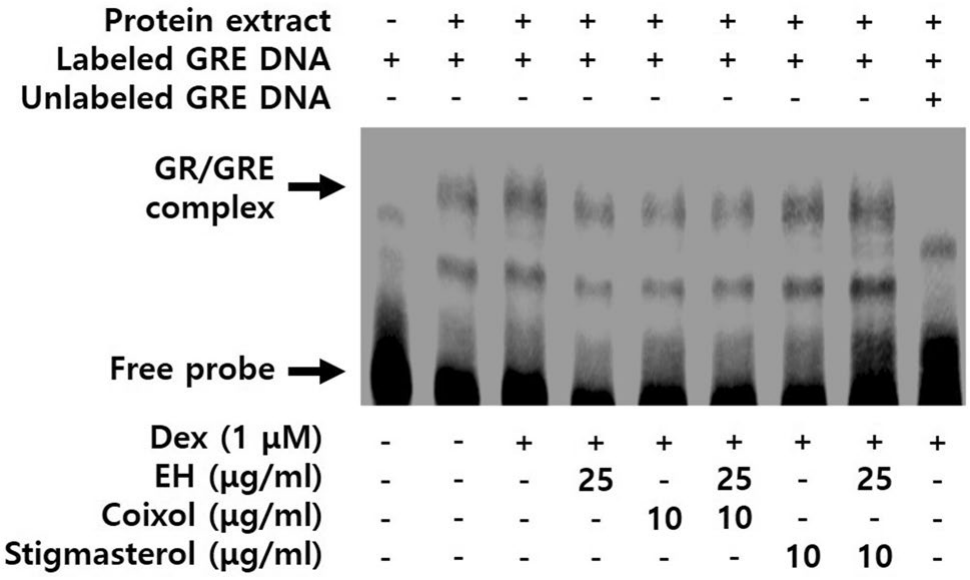
Coixol blocks nuclear GR binding activities in dexamethasone-induced 3T3-L1 cells. The EMSA method was used to determine nuclear GR-GREs binding activities using 10 µg of nuclear extract. *GR* glucocorticoid receptor, *EMSA* Electrophoretic mobility shift assays, *GRE* glucocorticoid response elements.

The Electrophoretic mobility shift assay (EMSA) was performed to investigate nuclear GR binding activities using 10 µg of nuclear extract. The image showed an upper shift of the band representing the interaction between GR-DNA complexes (GRE), which was intensified by the dexamethasone treatment (Fig. 7). EH and coixol decreased the nuclear GR-GRE interaction in dexamethasone-treated 3T3-L1 cells.

## Discussion

The present study provides several insights into how EH and CS efficiently collaborate in treating obesity. As mentioned earlier, the description of the molecular mechanisms of the EH-CS combination provided in our previous study was not sufficient. Therefore, we further performed bioinformatics analysis and in vitro experiments to gain a better understanding of the anti-obesity effects of the combination.

In traditional medicine, the stimulating effects of EH on the autonomic nervous system have been well-acknowledged and documented^29^. EH has been widely used in obesity management due to its effects such as the suppression of appetite, increase in metabolic rate, and improvement in exercise capacity^29,30^. However, US FDA banned the sales of all dietary supplements containing the herb ephedra and any ephedra group alkaloids (e.g. ephedrine, pseudoephedrine, etc.), due to reports of adverse effects such as hepatitis, nephritis, and cardiovascular toxicity^31^.

On the other hand, CS is used in several herbal prescriptions for metabolic diseases due to its well-established safety and efficacy profiles^32^. CS has been reported to have anti-diabetic, anti-hyperlipidemic, anti-oxidative, anti-cancer, and anti-inflammatory effects, which have been attributed to the presence of phytochemicals, such as phenols, flavonoids, triterpenes, and phytosterols^32,33^. Coixol and stigmasterol are constituent ingredients of CS and have been studied by several researchers to determine their pharmacological effects on various cellular targets related to metabolic diseases^32^. Coixol, which is regarded as a potent insulin secretagogue^34^, was reported to improve glucose tolerance and fasting blood glucose levels in diabetic animal models^35^. It has been shown to suppress nuclear factor kappa-light-chain-enhancer of activated B cells (NF-κB), mitogen-activated protein kinase (MAPK) pathways, and NOD-, LRR- and pyrin domain-containing protein 3 (NLRP3) inflammasome activation in lipopolysaccharide (LPS)-induced RAW264.7 cells^36^. Stigmasterol was reported to attenuate diabetic activity by targeting the glucose transporter protein type-4 (GLUT4) and alleviating nonalcoholic fatty liver disease (NAFLD) by altering the lipid metabolism in mice fed a high-fat western-style diet^37^. We expected that these compounds from CS may provide pharmacological properties distinct from the benefits of the EH treatment. However, this theory has not been investigated yet.

In the meantime, DEGs screened from the subcutaneous adipose tissue of morbidly obese patients overlapping with target lists of the EH-CS combination were used to investigate potential pathways and gene ontology annotations. The herbal combination was shown to be closely related to various metabolic pathways including ‘insulin signaling’, ‘AMPK signaling’, ‘cholesterol metabolism’, and ‘response to lipids’, particularly in the functional clusters of down-regulated DEGs (Fig. 1A and B). An interesting aspect of the analysis was that the PPI network of down-regulated DEGs was more likely comprised of genes that are targeted by the EH and CS combinations (yellow nodes), rather than EH alone, and this is reversed in the up-regulated PPI networks of DEGs (Fig. 1A, Fig. S4). The phenomenon of the unequal ratio of the EH-CS common target genes can be interpreted as follows: the CS might modulate down-regulated genes rather than the up-regulated genes in morbidly obese individuals. This suggests that CS may play a role in enhancing the signaling pathways which restore normal lipid metabolism in the obese. However, the theory has to be proved with evidence from experimental data.

We further evaluated the anti-adipogenic effects of the EH-CS combination in adipocyte differentiation. Markers for mechanism study of EH-CS was considered from key adipogenic genes and consisting genes of significant KEGG pathways or BP enrichment derived from analyses of down-regulated DEGs. The EH-CS treatment dose-dependently reduced intracellular lipids and regulated adipogenesis/lipogenesis-associated markers such as ACC, SREBP1, FAS, and C/EBPα through AMPK activation in differentiated 3T3-L1 cells. AMPK is one of the best-characterized anti-obesity targets. The treatment of metabolic diseases such as obesity, diabetes, and NAFLD includes the use of bioactive components that exert their beneficial effects via AMPK activation. The serine/threonine kinase AKT, also known as protein kinase B plays an essential role in adipogenesis at an early stage of adipocyte differentiation. Overexpression of AKT in 3T3-L1 cells has been known to promote glucose uptake and adipocyte differentiation^38^. Our results showed that EH-CS reduced lipid accumulation and inhibited adipocyte differentiation while it increased the phosphorylation level of AKT.

We hypothesized that some compounds in CS may enhance the pharmacological effect of EH. As the main compounds of EH accountable for weight-loss effect is already known (ephedrine and other analogue alkaloids), now it is crucial to elucidate major bioactive compound of CS. Hence, we tested the effects of two compounds of CS, identified in a previous study, on EH-treated adipocytes^25^. The EH extract, coixol, and stigmasterol treatments reduced the size and occurrence of lipid droplets in mature 3T3-L1 cells (Fig. 4). In most cases, the combination of EH with coixol or stigmasterol enhanced the effects of EH. Stigmasterol activated energy metabolism by the phosphorylation of AMPK and AKT. AKT plays a key role in multiple cellular processes as it regulates mitochondrial function and is an important therapeutic target in the treatment of diabetes and obesity^39^. However, coixol enhanced the efficacy of EH against the adiposity of 3T3-L1 cells without affecting the activity of AMPK. Coixol or stigmasterol reduced SREBP1, C/EBPα, and FASN when combined with EH.

Overall, the EH extract alone was able to increase or activate energy metabolism via the AMPK pathway, as was demonstrated by the increased phosphorylation ratio of AMPK or ACC (Figs. 4 and 5). However, EH alone had minimal effects on the regulation of adipogenic markers (SREBP, C/EBP, FASN, and PPARγ) in repeated experiments (Fig. 5A,C). The impact of EH treatment on those markers is consistent with that seen in some markers in the previous study (p-AMPK, PPARγ, or C/EBPa), but not in other markers (FASN or SREBF1). This may be attributed to the different time-point of the mRNA harvest^25^.

Strikingly, positive effects were observed with the co-treatment of the EH extract and CS compounds via the broad markers of adipogenesis/lipogenesis as demonstrated by the mature 3T3-L1 adipocyte model (Figs. 4 and 5). Specifically, between the two CS-derived compounds, coixol showed better effects as compared to stigmasterol. As per our results, coixol can be tentatively seen to be a major partner component that works in combination with EH.

Also, the GR (official gene name: *NR3C1*) was found to be an obesity-related common target for both EH and CS and placed in the first functional cluster in down-regulated DEGs as demonstrated by the bioinformatics analysis (Fig. 1A). Based on the frequent appearance of the gene from the top-enriched BP ontologies, derived from the functional cluster analysis, GR was expected to have a significant role in the action of the EH-CS combination. EH, Coixol, and stigmasterol, when used either alone or in co-treatments, inhibited the dexamethasone-induced GR translocation to the nucleus in 3T3-L1 adipocytes and the efficacy was the highest in the case of the EH with coixol co-treatment (Fig. 6).

Our data allow us to hypothesize that EH and the two compounds of CS may have different effects on adipocytes. EH with coxiol or stigmasterol can antagonize GR, alter lipid metabolism through reduced lipogenesis and increased lipolysis, and inhibit the development of insulin resistance in mature adipocytes. Furthermore, the combination of EH with coixol or stigmasterol shows anti-obesity effects by reducing the expression of adipogenic transcription factors such as C/EBPα and PPARγ through the successful inhibition of GR activity as transcription factors (TFs) in the nucleus. The combination of EH and CS could thus be proposed as a therapeutic option for the treatment of obesity. Further investigation is therefore warranted.

## Methods

### Chemicals

Dulbecco’s Modified Eagle’s Medium (DMEM) was purchased from Hyclone (Logan, UT, USA), and penicillin/streptomycin solution and fetal bovine serum (FBS) were purchased from Invitrogen (Carlsbad, CA, USA). 3-Isobutyl-1-methylxanthine (IBMX) and other reagents were purchased from Sigma Aldrich (St. Louis, MO, USA), and used for cell differentiation and maturation. The EZ-Cytox assay kit was obtained from the Daeil Lab Service (Chungcheongkuk-do, South Korea). The phosphorylated or non-phosphorylated primary antibodies of AMPK, AKT, ACC, C/EBPα, SREBP1, and GR were purchased from Cell Signaling Technology (Berkeley, CA, USA). Lamin B and β-actin were obtained from Santa Cruz Biotechnology (Santa Cruz, CA, USA), which also supplied secondary antibodies. The oligonucleotide primers for real-time qPCR were produced by Macrogen (Seoul, South Korea).

### Human microarray data acquisition and processing

The microarray dataset of women of obese and non-obese phenotype (accession number: GSE59034) was accessed via the GEO database (https://www.ncbi.nlm.nih.gov/geo/query/acc.cgi?acc=GSE59034) of the National Center of Biotechnology Information (NCBI). The dataset comprised of subcutaneous white adipose tissue from morbidly obese female patients (average BMI score>40, data obtained prior to bariatric surgery) and non-obese female counterparts (16 in each group, total 32). The microarray dataset is based on the GPL11532 platform (HuGene-1_1-st) Affymetrix Human Gene 1.1 ST Array [transcript (gene) version].

### Screening of morbid obesity-related DEGs and construction of PPI networks

GEO2R is an online interactive web tool that can be utilized to screen DEGs from the microarray dataset by comparing at least two groups of samples assigned by users (http://www.ncbi.nlm.nih.gov/geo/geo2r/). GEO2R identifies DEGs from the dataset using the GEO query which links the GEO data repository and the R/Bioconductor equipped with the Limma R package^40,41^. Genes were considered as DEGs with their adjusted p-values (p < 0.05) and fold change (|log2(fold change)|> 1). Meanwhile, traditional Chinese medicine systems pharmacology database (TCMSP, https://old.tcmsp-e.com/tcmsp.php (accessed on 20, July, 2022))^42^ was used to search potential targets of EH and CS combination. Then the DEGs were compared with the potential target lists of EH-CS and overlapping genes were categorized to be analyzed in further study.

The PPI networks between the up-regulated and down-regulated DEGs were separately constructed using the Search Tool for the Retrieval of Interacting Genes (STRING database, https://string-db.org/)^43^ and visualized after importing them into the Cytoscape software (Version 3.91)^44^. The functional cluster analysis of each DEG PPI network was performed with the Cytoscape MCODE plugin to identify significant clusters^45^.

### GO and KEGG pathway enrichment analyses

The enrichment analysis of the gene ontology (GO) and the Kyoto encyclopedia of genes and genomes (KEGG) pathway^46^ based on each up-regulated or down-regulated DEG was performed in the STRING database. The results of the analysis were exported and processed to be visualized as bubble plots created by the ggplot R package with the public script^47^.

### Preparation of samples

Dried herbs of Ephedrae herba and Coicis semen were purchased from Herbmaul (Chungcheongbuk-do, South Korea). We used identical samples obtained from previous study^48^. To brief the extraction process of EH-CS or EH extracts, the Ephedrae herba and Coicis semen in a 1:1 combination (50 g:50 g) or Ephedrae herba (100 g) were ground to a powder and extracted with 1 L distilled water at 95 ℃ for 1 h. The crude extracts were filtered through an 8 μm-pore-size Whatman filter paper, concentrated by rotary evaporation (Buchi, Flawil, Switzerland) at 95 ℃, and freeze-dried to obtain the lyophilized extracts of EH-CS, which were eluted with Dulbecco’s phosphate buffered saline (DPBS) and filtered through a 0.22 μm syringe filter before use.

### Cell culture and treatment

3T3-L1 preadipocytes (ATCC CL-173) were obtained from the Korea Cell Line Bank (KCLB, Seoul, South Korea). The 3T3-L1 preadipocytes were cultured in DMEM supplemented with 10% fetal bovine serum, and 1% penicillin/streptomycin at 37 ℃ in a humidified 5% CO_2_ atmosphere. To evaluate the anti-adipogenic effects, the 3T3-L1 preadipocytes were seeded on 12-well plates at 2 × 10^5^ cells per well in DMEM supplemented with 10% FBS and incubated to full confluence and then for an additional 2 days. Differentiation was initiated by exchanging the medium with the differentiation medium (DMEM supplemented with 10% FBS, 1 μM of dexamethasone, 0.5 mM of 3-isobutyl-1-methylxanthine, 10 μg/ml of insulin) for 3 days, and the cells were further incubated in the maturation medium (DMEM supplemented with 10% FBS containing 10 μg/ml insulin) with the samples (EH-CS, EH, coixol and stigmasterol) for 12–24 h (for real-time PCR, Western blot, and Immunofluorescence microscopy) or 8 days (for ORO staining), and the maturation medium was exchanged every two days.

### Cell viability assessment

The cell viability of the 3T3-L1 cells was determined using the EZ-Cytox assay kit (Daeil Lab Service, Chungcheongbuk-do, South Korea) according to the manufacturer's instructions. Briefly, Cells were seeded in 96-well plates in FBS-free DMEM at 2 × 10^3^ cells per well and then incubated with the samples for 24 h. The optical densities (ODs) of the reactants were measured at 450 nm using a microplate spectrophotometer (Versamax, Molecular Devices, CA, USA).

### Oil red O staining

Lipid accumulation was assessed by ORO staining. In brief, mature 3T3-L1 cells were washed with DPBS, fixed with 10% formalin for 1 h at room temperature, washed once with 60% isopropanol, and air-dried. A stock solution of ORO was prepared by filtering a solution of 0.175 g of ORO powder in 50 ml of isopropanol and diluting the filtrate with distilled water in a ratio of 3:2. Cells were stained with the ORO solution for 15 min, washed three times with distilled water, air-dried, and examined under an inverted microscope system equipped with a camera (DMI 6000, Leica, Wetzlar, Germany). For quantitative analysis, the stains were re-dissolved in isopropanol, and the absorbances were measured at 520 nm using a spectrophotometer (VersaMax, Molecular Devices, CA, USA).

### Quantitative real-time polymerase chain reaction

The expression levels of adipogenesis/lipogenesis-associated genes were determined by qPCR. The total RNA was isolated from the 3T3-L1 cells using the Trizol reagent (Invitrogen, Carlsbad, CA, USA), according to the manufacturer’s instructions. Briefly, reverse transcription was performed using an AccuPower RT PreMix (Bio-neer, Daejeon, South Korea) and oligo (dt) 18 (Invitrogen, Carlsbad, CA, USA). Primer-specific binding cDNA was amplified on a Light Cycler 480 PCR system (Roche, Basel, Switzerland) using 10 μl of SYBR green Master mixture (Roche, Switzerland), 8 μl of ultrapure water, 10 pmol/μl of primer, and 1 μl of template cDNA. Amplification was performed using the following schedule: denaturation at 95 ℃ for 3 min, followed by 45 amplification cycles (denaturation at 95 ℃ for 10 s, annealing at 55–60 ℃ for 10 s and 72 ℃ for 20 s). Threshold cycle values (Ct value) were used to quantify the PCR products. Relative expression levels were calculated by dividing gene Ct values by that of β-actin. All data were acquired using a LightCycler 480 instrument and software. The Primers used are described in the Supporting Information Table (Supplementary Table 1).

### Western blot analysis

The levels of adipogenesis/lipogenesis-associated markers were determined by the Western blot assay. Briefly, cells were washed with DPBS and lysed with radioimmunoprecipitation assay (RIPA) buffer (Thermo Fisher Scientific, Rockford, IL, USA) containing a protease and phosphatase inhibitor cocktail (Gendepot, Barker, TX, USA). Protein concentrations were estimated using the BCA kit (Thermo Fisher Scientific, Rockford, IL, USA). Equal amounts of proteins (30 μg) mixed with the Lane Marker Reducing sample buffer (Thermo Scientific, Rockford, IL, USA) were loaded into 10% sodium dodecyl-sulfate polyacrylamide gel electrophoresis (SDS‐PAGE) gels, electrophoresed, and transferred to polyvinylidene difluoride (PVDF) membranes (Merck, Minneapolis, MN, USA) at 100 V for 60 min using an electrophoretic transfer cell (Bio-rad, Hercules, CA, USA). Membranes were blocked with 5% BSA in TBS/T (TBS containing 0.1% Tween 20) for 2 h at room temperature, and the blots were incubated with primary antibodies (diluted at 1:1000 in TBS/T containing 3% BSA) overnight at 4 ℃ with gentle shaking. After washing with TBS/T, membranes were incubated with secondary antibodies (diluted at 1:3000 in TBS/T containing 1% BSA) at room temperature for 2 h. Chemiluminescent blots were developed using an Enhanced Chemiluminescence (ECL) buffer (Super Signal West Pico, Thermo Fisher Scientific), and images were captured using a Western blot imaging system (Fusion Solo, Vilber Lourmat, Collegien, France).

### Immunofluorescence microscopy

To trace the nuclear translocalization of the GR and SREBP1 proteins, the 3T3-L1 cells were incubated on Lab-Tek II chamber slides (Nunc, IL, USA). Briefly, the cells were washed with DPBS and fixed with 4% formaldehyde solution for 10 min, permeabilized with 0.1% Triton X-100 for 10 min, blocked with 1% BSA for 1 h, and labeled with 2 μg/ml of primary antibody overnight at 4 ℃. Cells were washed twice with DPBS and labeled with 2 μg/ml of fluorescein isothiocyanate (FITC) and tetramethylrhodamine isothiocyanate (TRITC) secondary antibody for 45 min at room temperature. Cells were washed twice with DPBS and stained using a mounting medium containing 4’,6-diamidino-2-phenylindole (DAPI, Vector Laboratories, CA, USA). Fluorescence images were captured under a fluorescence microscope (BX50, Olympus, Japan).

### Preparation of nuclear and cytosolic fractions

Nuclear and cytosolic proteins were separated using a nuclear and cytoplasmic extraction kit (Thermo Fisher Scientific, Rockford, IL, USA). Briefly, 3T3-L1 cells were seeded on 6-well plates at 4 × 10^5^ cells per well in DMEM supplemented with 10% FBS and incubated to full confluence (100%) and then for a further 2 days. Differentiation was initiated by exchanging the medium with the differentiation medium for 3 days, and the cells were further incubated in the maturation medium for 8 days. Cells were stimulated with dexamethasone (1 μM) and samples for 12 h. The nuclear translocation of the GR and SREBP1 proteins from the cytoplasm was assessed by the Western blot assay.

### EMSA

To obtain nuclear proteins, 3T3-L1 cells were cultured in plates until the cells reached 100% confluence. The cells were treated with the sample and dexamethasone for 3 h. The cells were washed with ice-cold PBS, harvested, and nuclear proteins were extracted using the Nuclear and Cytoplasmic Extraction Reagents kit (Thermo Fisher Scientific, Rockford, IL, USA). Protein concentrations were determined using the BCA kit. Oligonucleotide sequences with 5′ end biotin-labeled and unlabeled glucocorticoid response elements (GREs) primers were purchased from Macrogen (Seoul, South Korea). The Light Shift Chemiluminescent electrophoretic mobility shift assay (EMSA) Kit, (Thermo Fisher, MA, USA) was used according to the manufacturer’s instructions in subsequent steps. Briefly, binding buffer, poly (dI·dC), and the 5′-biotin labeled probe were incubated with 10 μg of nuclear proteins in a volume of 20 μl. For the competition assay, the unlabeled probe was incubated at room temperature for 20 min before adding the labeled probe. A 5X loading buffer was added to each tube before loading. The DNA–protein complexes were separated on a 7% non-denaturing polyacrylamide gel, with a constant voltage of 100 V in a tris–borate-EDTA (TBE) buffer and transferred to a positive charge Biodyne B nylon membrane (Thermo Fisher, MA, USA) at 70 V for 30 min using an electrophoretic transfer cell. After the transfer was completed, the membrane was cross-linked and the biotin-labeled DNA was detected using a chemiluminescent detection reagent. The images were captured using a western blot imaging system (Fusion Solo, Vilber Lourmat, Collegien, France).

### Statistical analysis

The experimental data were analyzed using the Graph Pad Prism version 5.0 software (Graph Pad, La Jolla, CA, USA). Standard curves were constructed using Excel and PowerPoint (Microsoft, Redmond, WA, USA). Analysis of variance and One-Way ANOVA with Dunnett’s multiple comparison tests were used to determine the significance of the differences. Results are presented as means ± SDs, and p-values of < 0.05 were considered statistically significant.

### Supplementary Information

Supplementary Information 1.

Supplementary Information 2.

Supplementary Information 3.

Supplementary Information 4.

## Data Availability

The datasets generated and/or analysed during the current study are available in the GEO database repository, (https://www.ncbi.nlm.nih.gov/geo/), GEO2R repository, (http://www.ncbi.nlm.nih.gov/geo/geo2r/), TCMSP repository, (https://old.tcmsp-e.com/tcmsp.php), and STRING repository, (https://string-db.org/).

## Abbreviations

AMPK: AMP-activated protein kinase
AKT: Protein kinase B
ACC: Acetyl coenzyme A carboxylase
C/EBPα: CCAAT/enhancer binding protein α
SREBP1: Sterol regulatory binding protein 1
PPARγ: Peroxisome proliferator-activated receptor gamma
IRS1: Insulin receptor substrate 1
FASN: Fatty acid synthase
EH: Ephedrae herba
CS: Coicis semen
GR: Glucocorticoid receptor
GREs: Glucocorticoid response elements
DEG: Differentially expressed genes
NF-κB: Nuclear factor kappa-light-chain-enhancer of activated B cells
MAPK: Mitogen-activated protein kinase
NLRP3 inflammasome: NOD-, LRR- and pyrin domain-containing protein 3
LPS: Lipopolysaccharide
NAFLD: Nonalcoholic fatty liver disease
GLUT4: Glucose transporter protein type-4
