# Supplementary material for: The combination of Ephedrae herba and coixol from Coicis semen attenuate adiposity via glucocorticoid receptor regulation
Source: Sci Rep. 2023 Nov 21;13:20324. doi: 10.1038/s41598-023-47553-3 (PMC10663538; doi:10.1038/s41598-023-47553-3)

**Supplementary Data**

**The combination of Ephedrae herba and coixol from Coicis semen attenuate adiposity via glucocorticoid receptor regulation**

**Supplementary Table 1: Primer sequence used in this study.**

| **Genes** | **Sequence (5’-3’)** | | **Tm (˚C)** |
| --- | --- | --- | --- |
| **SREBF1** | Forward | *5’-* GGA ACA GAC ACT GGC CGA *-3’* | 58.4 |
|  | Reverse | *5’-* AAG TCA CTG TCT TGG TTG TTG AT *-3’* | 59.3 |
| **CEBPA** | Forward | *5’-* GCG CAA GAG CCG AGA TAA AG *-3* | 60.5 |
|  | Reverse | *5’-* CAC GGC TCA GCT GTT CCA *-3’* | 58.4 |
| **FASN** | Forward | *5’-* CCT CCA AGA CTG ACT CGG *-3’* | 58.4 |
|  | Reverse | *5’-* CAG TGT GCT CAG GTT CAG TT *-3’* | 58.4 |
| **PPARG** | Forward | *5’-* AGT GAC TTG GCT ATA TTT ATA GCT GTC ATT *-3’* | 65.3 |
|  | Reverse | *5’-* TGT CTT GGA TGT CCT CGA TGG *-3’* | 61.3 |
| **GRE** | Forward | *5’-* CAC CAG GAC ACA CTC TGT CCT CTC TTA *-3’* | 66.6 |
|  | Reverse | *5’-* TAA GAG AGG ACA GAG TGT GTC CTG GTG *-3’* | 66.6 |
| **β-Actin** | Forward | *5’-* GAC GGC CAG GTC ATC ACT ATT G *-3’* | 64.0 |
|  | Reverse | *5’-* CCA CAG GAT TCC ATA CCC AAG A *-3’* | 62.1 |

Figure S1 Validation of selected gene expression profiles from GSE59034. Total 32 samples (16 obese patients and 16 non-obese counterparts) were included. (A) Boxplot of selected samples. (B) Venn diagram of DEGs between two groups. (C) Adjusted p-value histogram of all analyzed genes. (D) UMAP plot of all 32 samples. (E) Volcano plot visualizing the distribution of DEGs in obese group by their fold change and p-value (adjusted p-value < 0.05 and |log2FC| > 1).


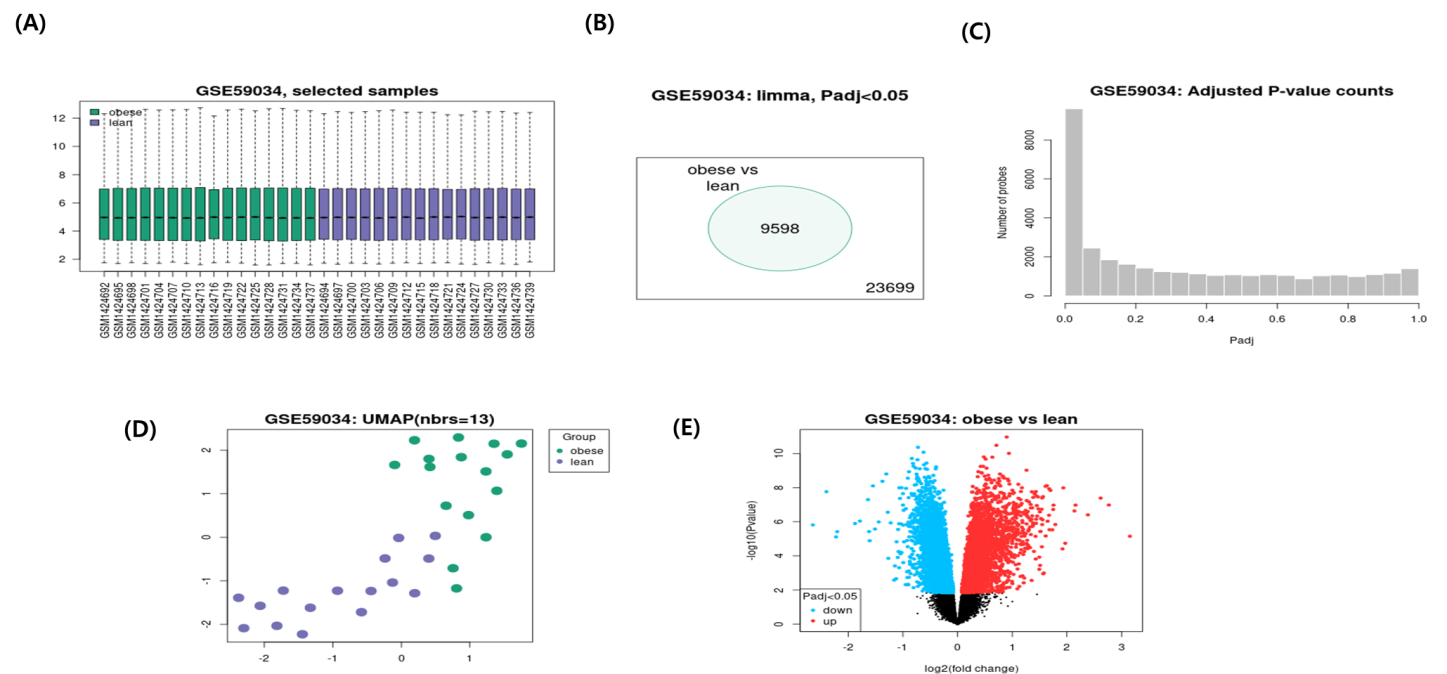


Figure S2 Venn diagram visualizing the distribution of up- or down-regulated DEGs overlapping with potential targets of EH and CS.


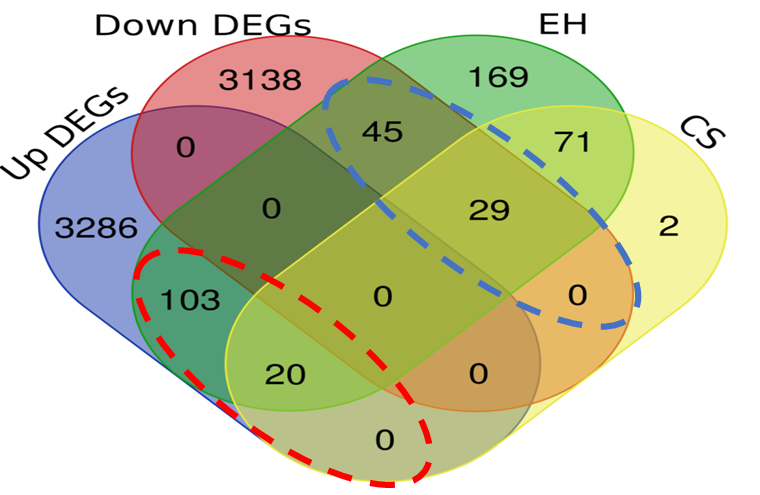


Figure S3 Total protein-protein interaction network of EH-CS combination divided by (A) up-regulated or (B) down-regulated gene lists.


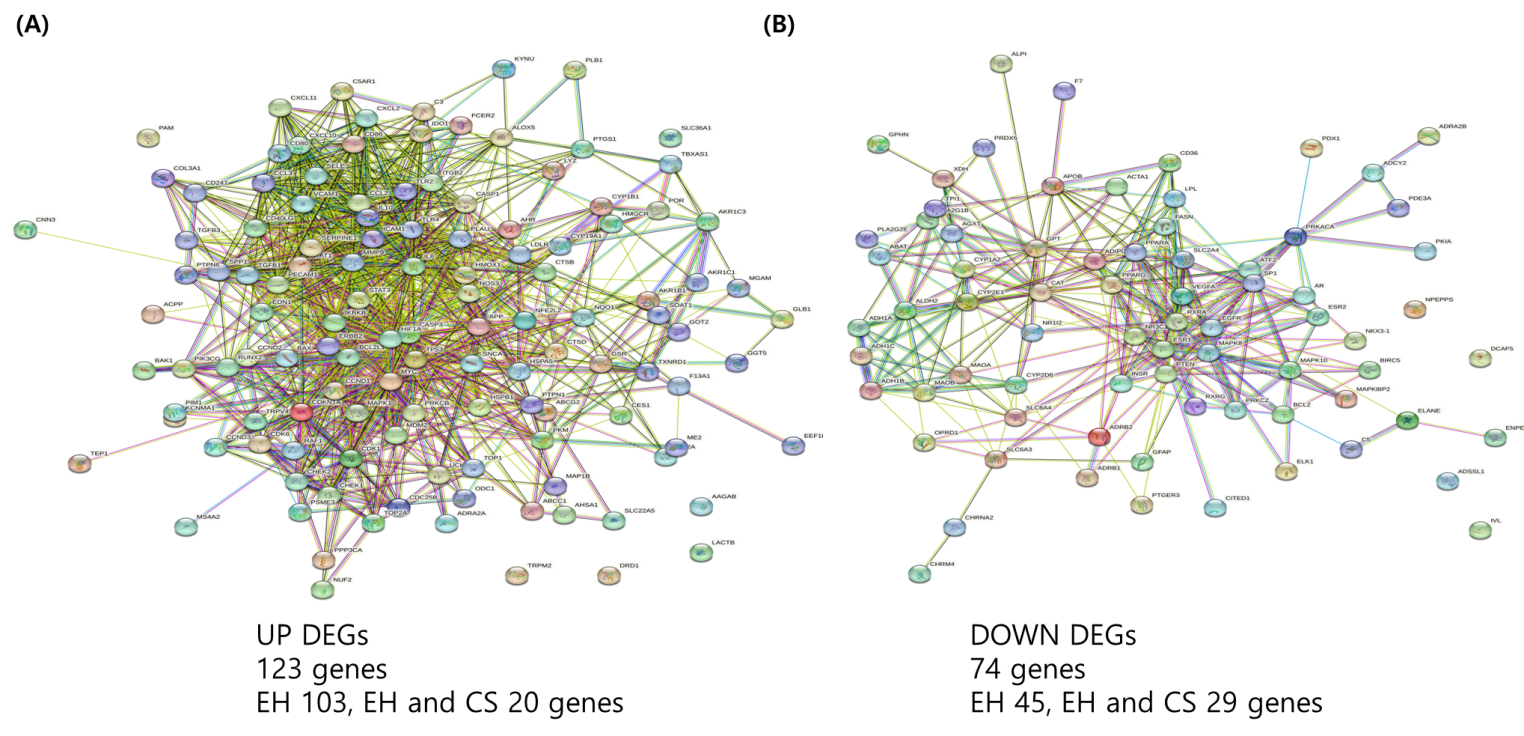


Figure S4 Functional cluster analysis of all up-regulated DEGs for EH-CS combination.


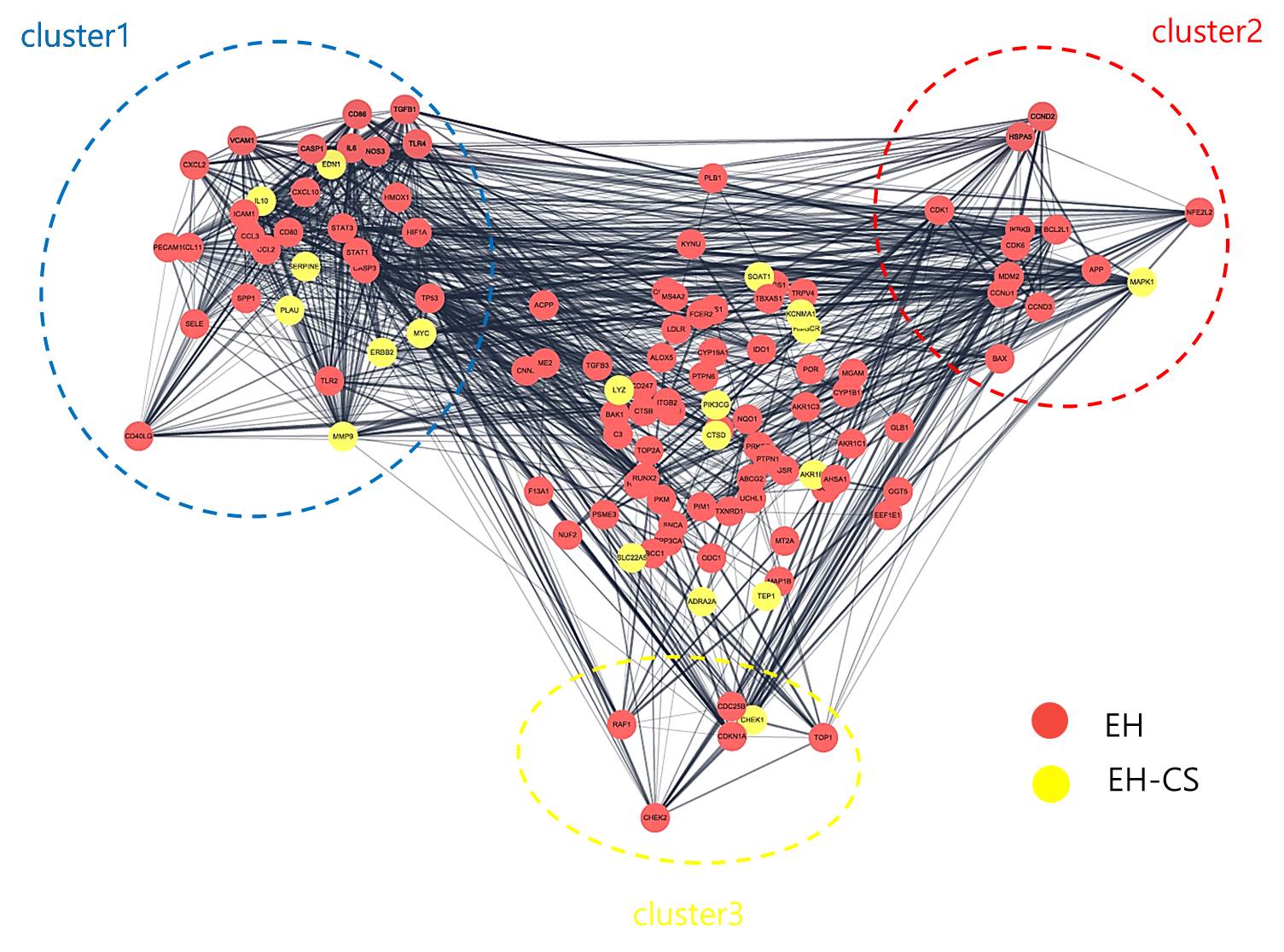

Supplement: Supplementary file 1 — Supplementary Information 1. [file 41598_2023_47553_MOESM1_ESM.docx]
