# Supplementary material for: The combination of Ephedrae herba and coixol from Coicis semen attenuate adiposity via glucocorticoid receptor regulation
Source: Sci Rep. 2023 Nov 21;13:20324. doi: 10.1038/s41598-023-47553-3 (PMC10663538; doi:10.1038/s41598-023-47553-3)

The combination of Ephedrae herba and coixol from Coicis semen attenuate adiposity via glucocorticoid receptor regulation

Figure 2 (E)

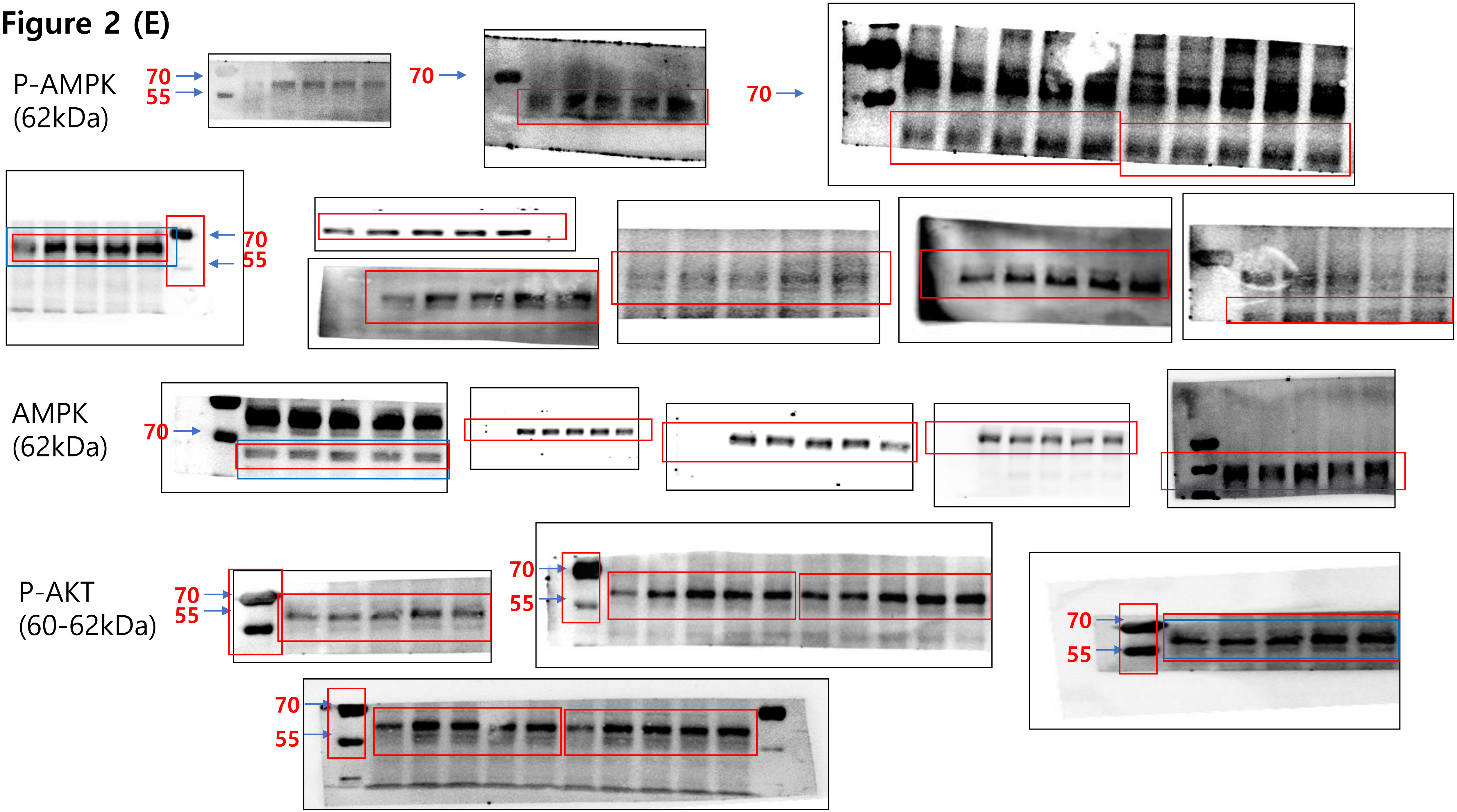
P-AMPK
(62kDa)
70
55
70
70
70
55
AMPK
(62kDa)
70
P-AKT
(60-62kDa)
70
55
70
55
70
55
70
55

Figure 2 (E)   Figure 3 (B)

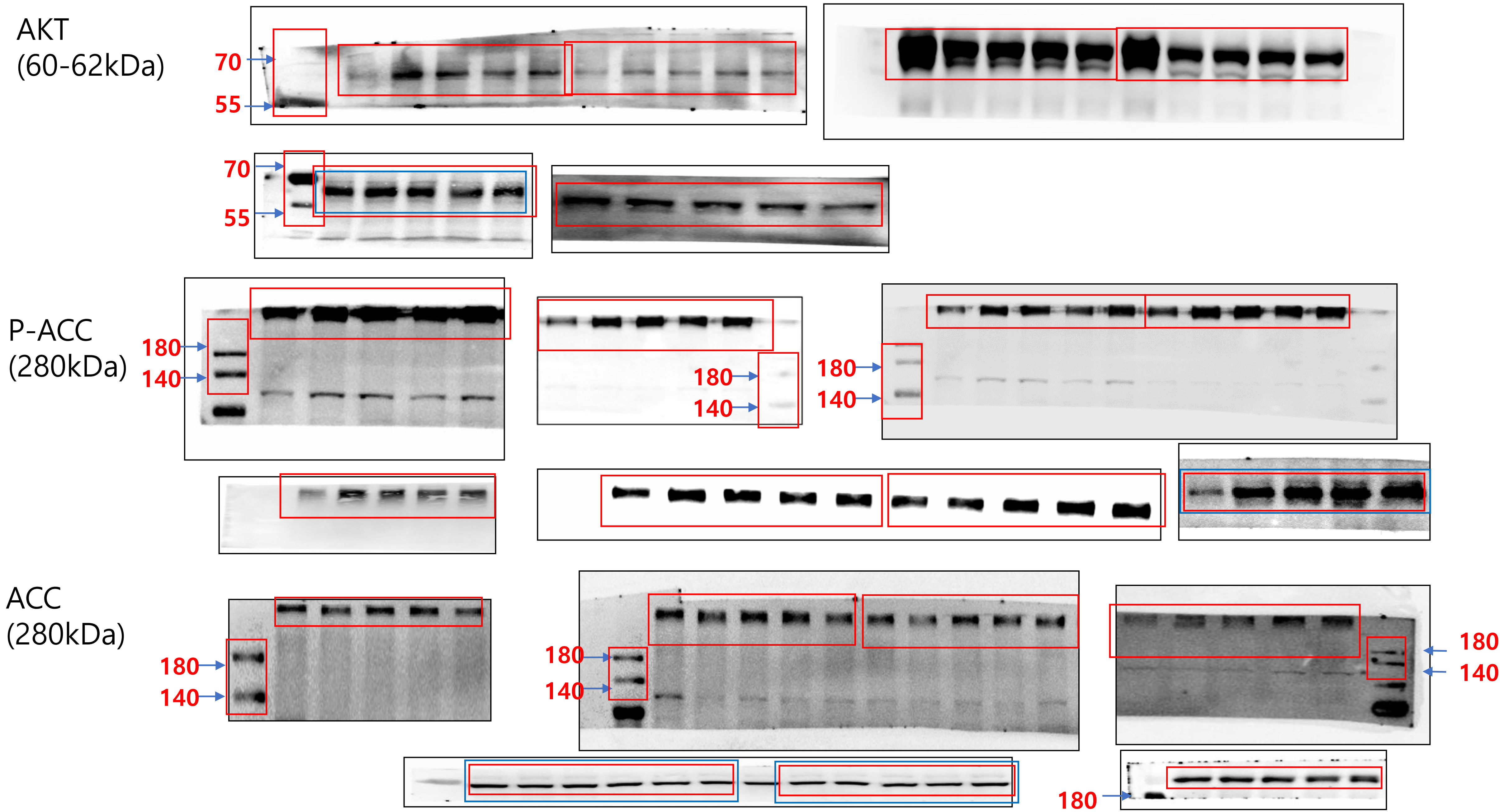

Figure 2 (E) Figure 3 (B)

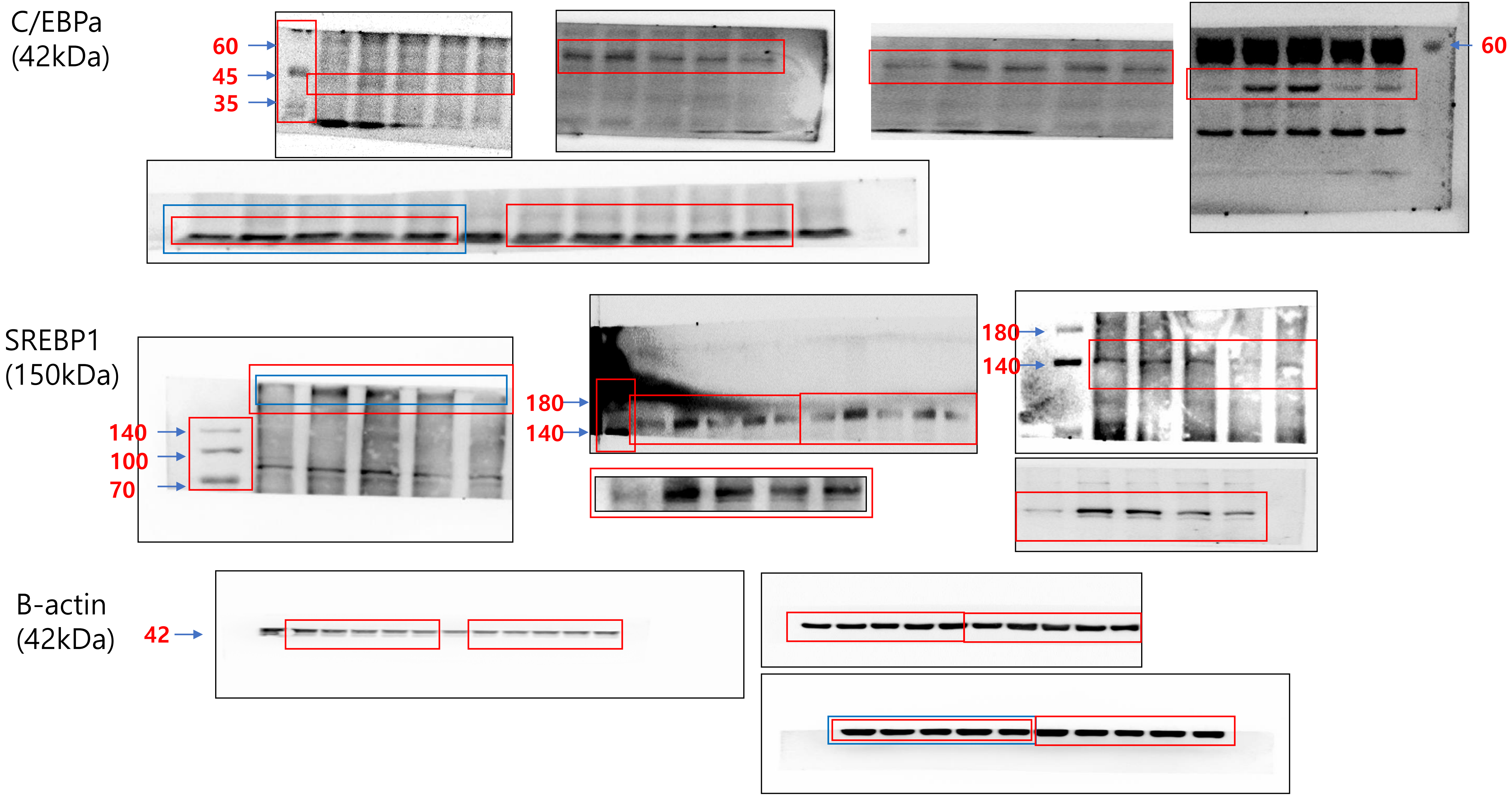

Figure 4 (D)

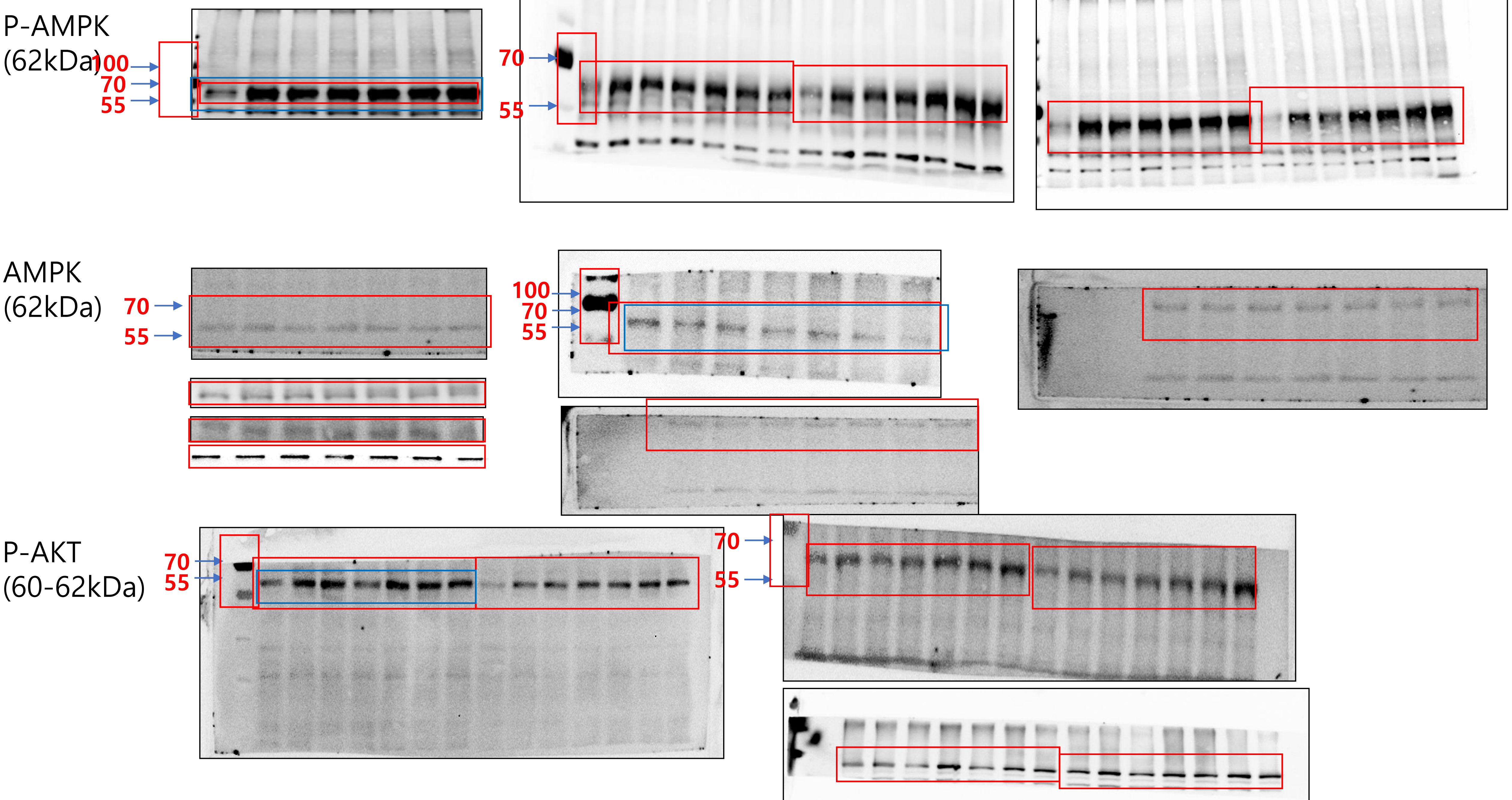
P-AMPK
(62kDa)
100
70
55
70
55
AMPK
(62kDa)
70
55
100
70
55
P-AKT
(60-62kDa)
70
55
70
55

Figure 4 (D) Figure 5 (B)

AKT
(60-62kDa)

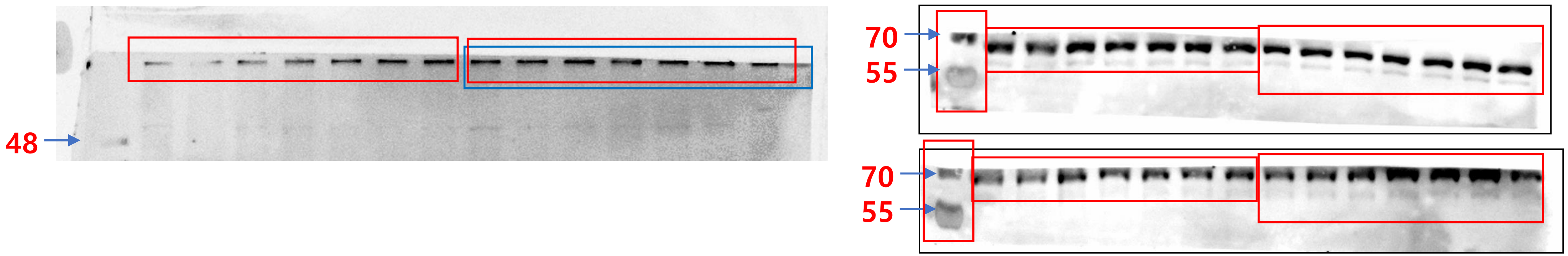

P-ACC
(280kDa)

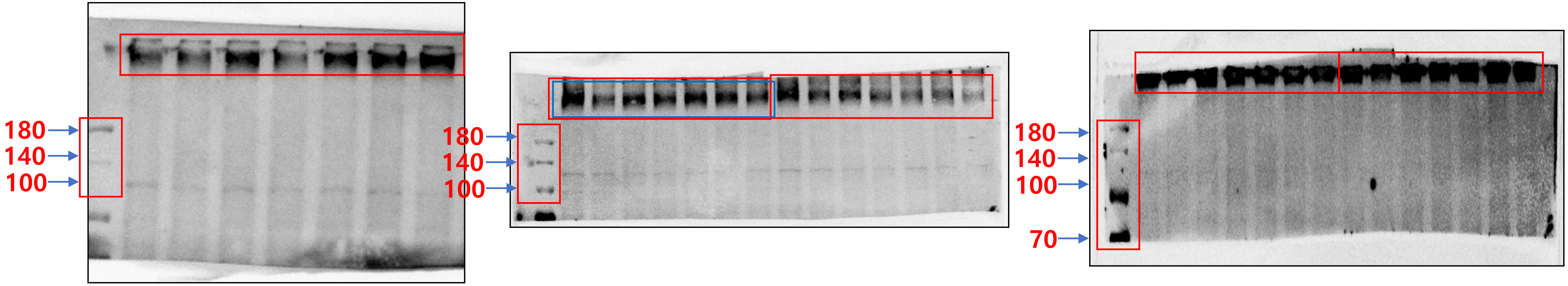

ACC

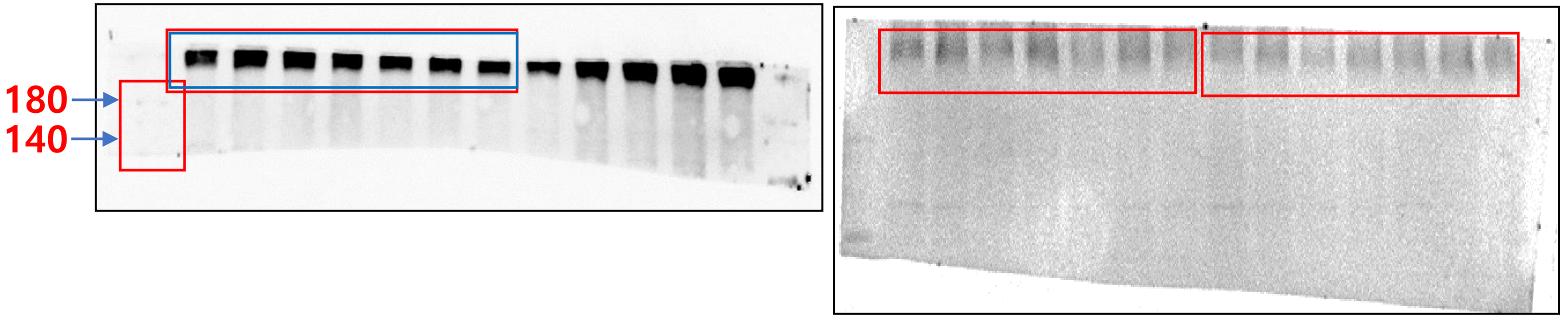

Figure 4 (D) Figure 5 (B)

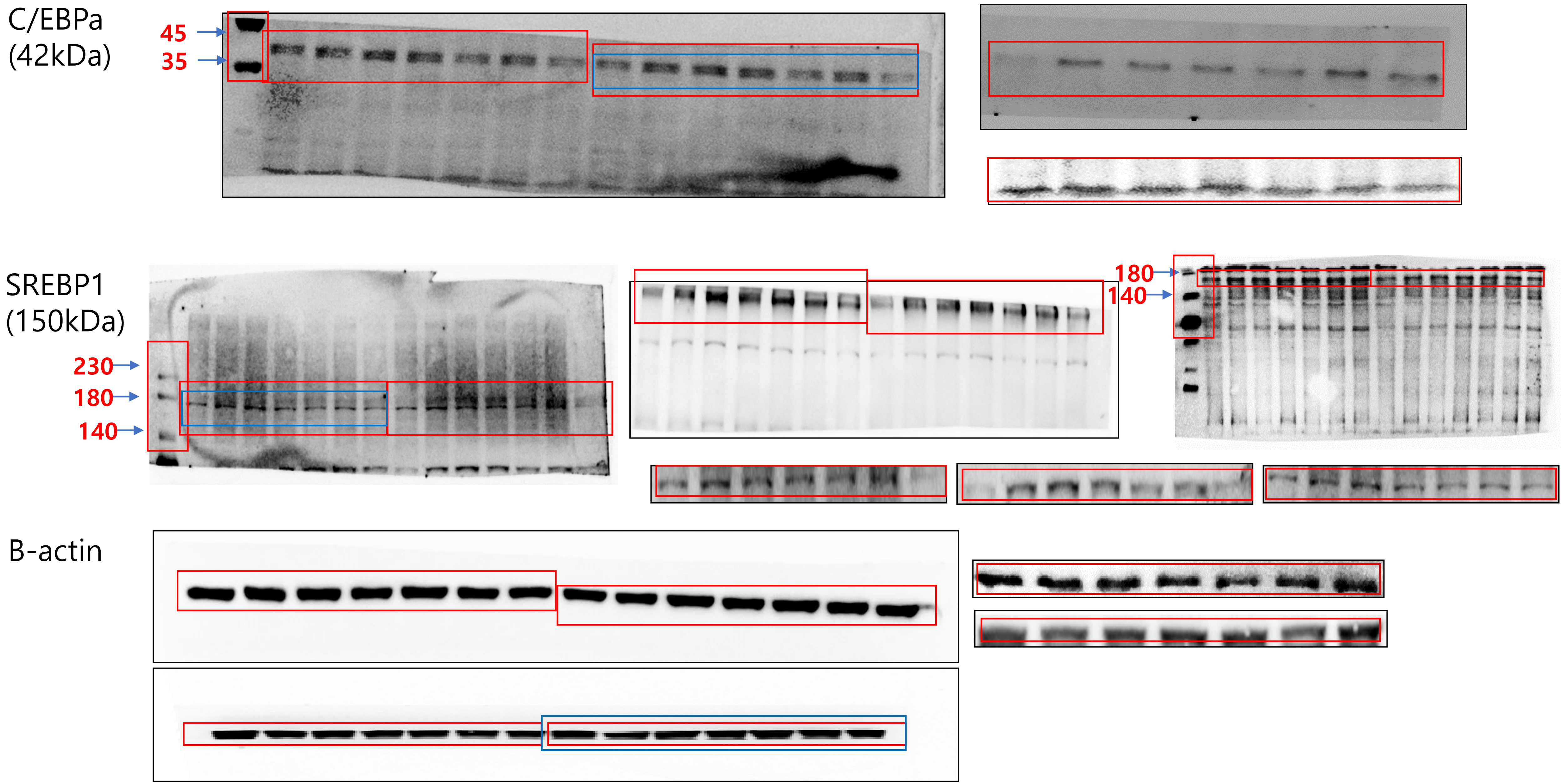

Figure 6 (B)

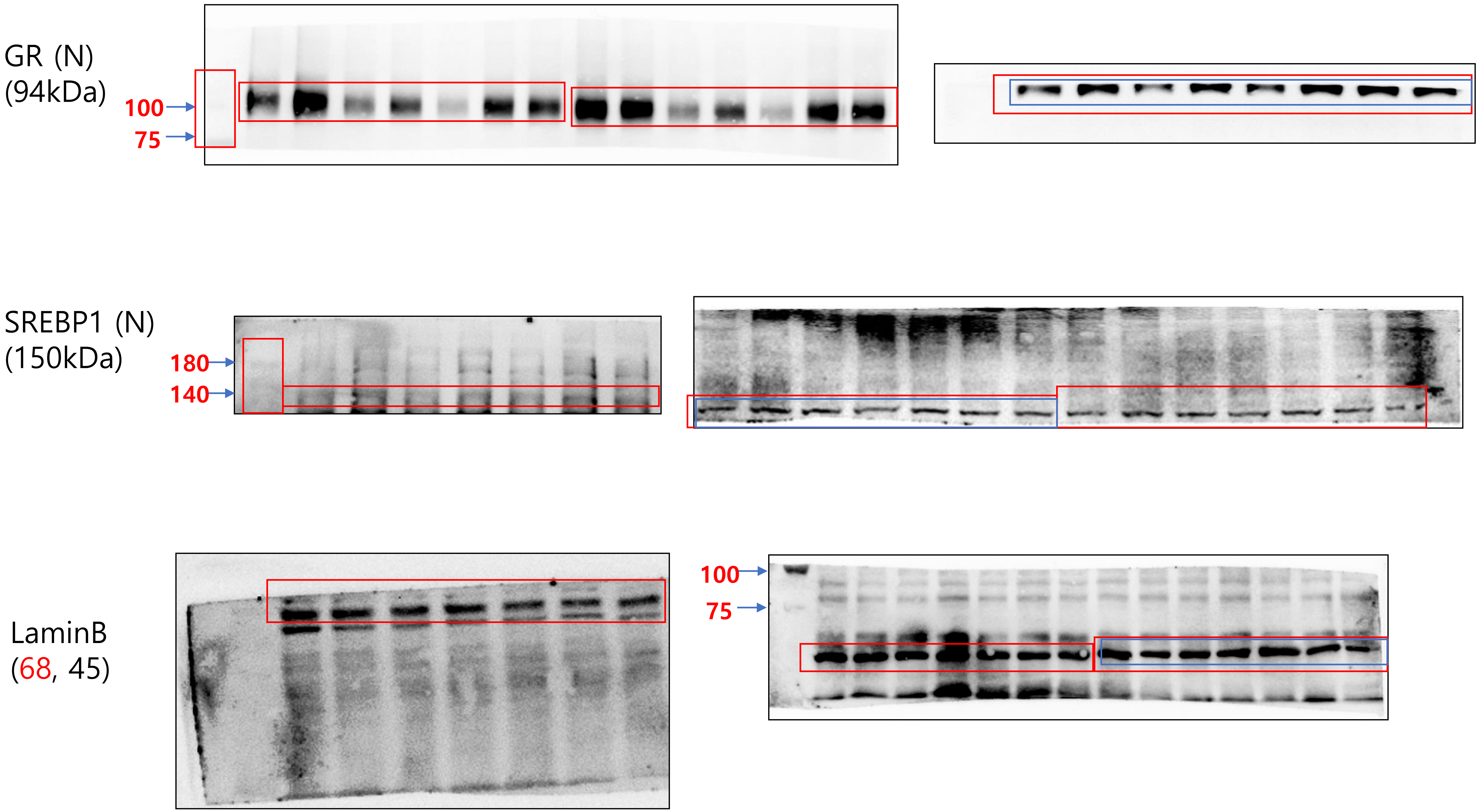

Figure 6 (B)

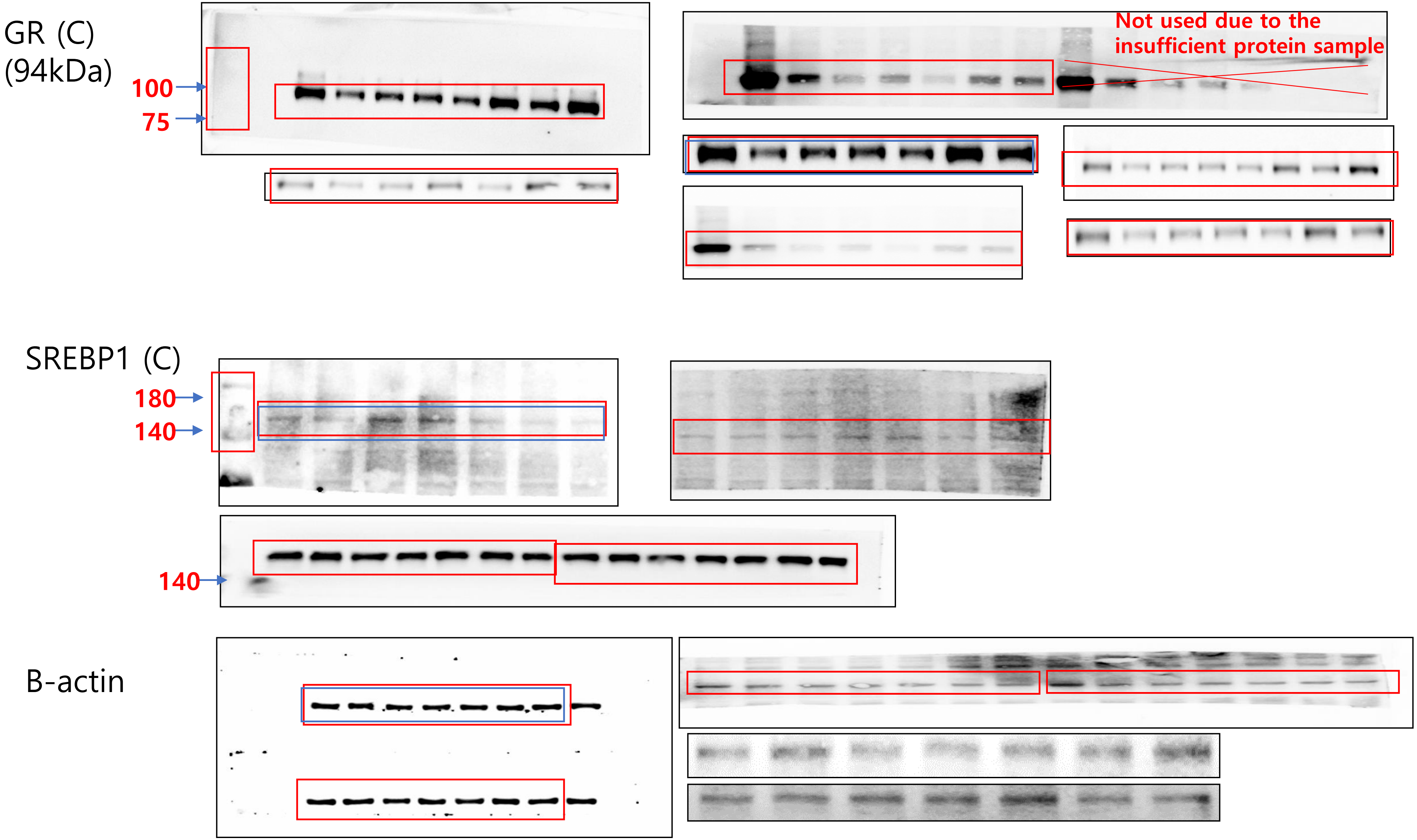
GR (C)
(94kDa)
100
75
Not used due to the
insufficient protein sample
SREBP1 (C)
180
140
140
B-actin

Figure 7

EMSA image used in Figure 7

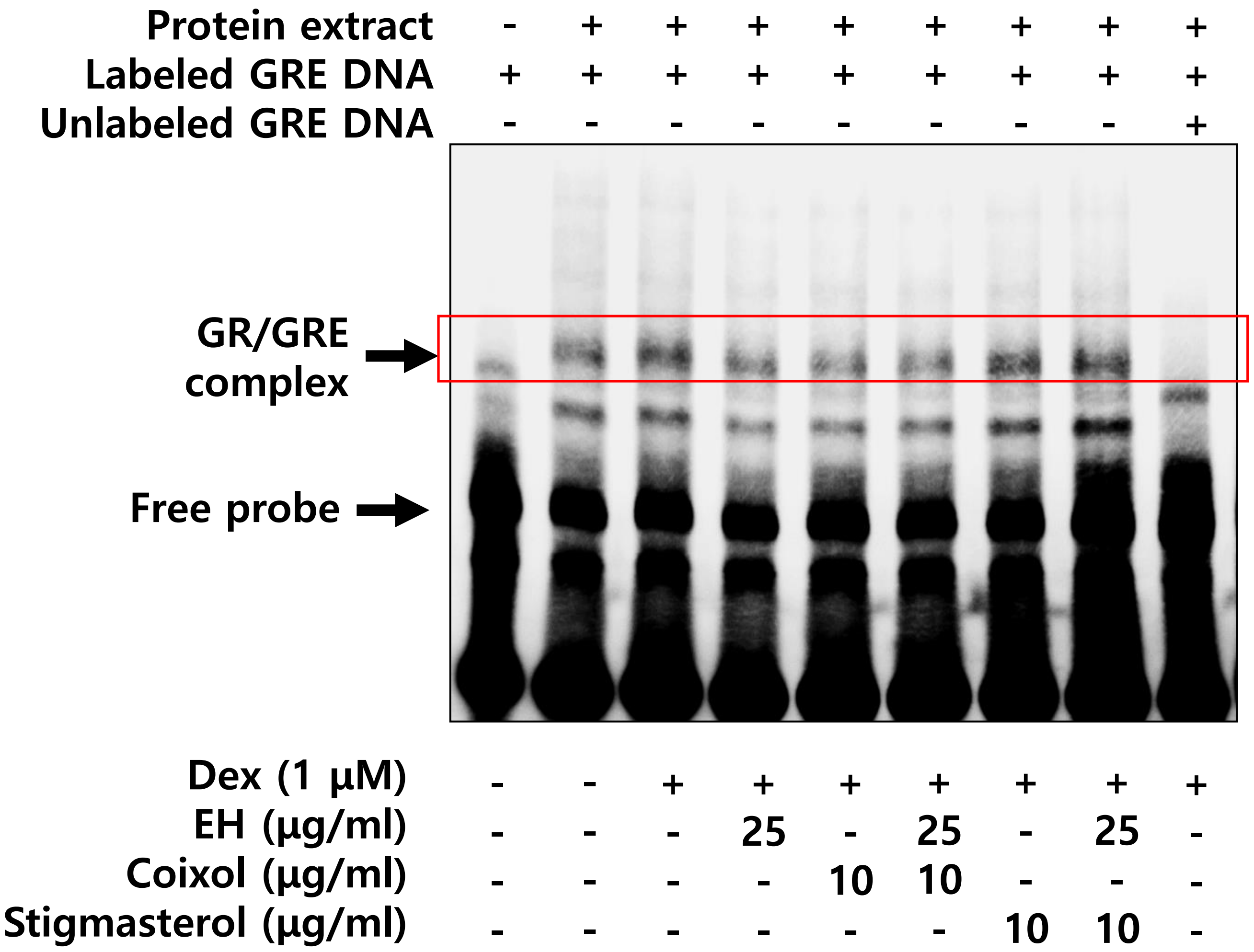

Same EMSA blot

Images obtained with different exposure time

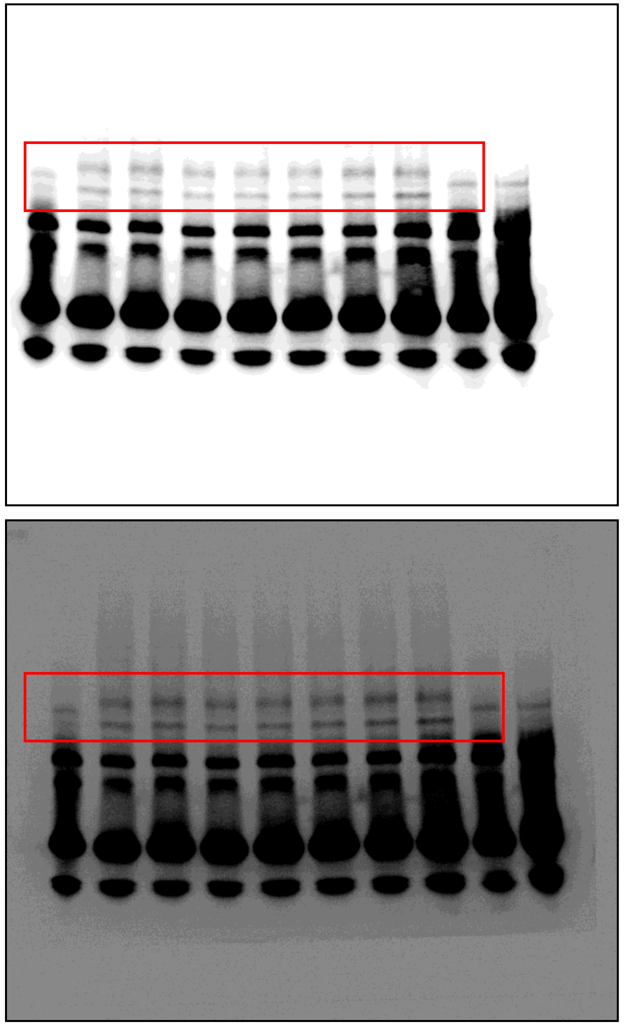

Supplement: Supplementary file 2 — Supplementary Information 2. [file 41598_2023_47553_MOESM2_ESM.pdf]
